# Pectobacterium carotovorum Phage vB_PcaM_P7_Pc Is a New Member of the Genus *Certrevirus*

**DOI:** 10.1128/spectrum.03126-22

**Published:** 2022-11-08

**Authors:** Kishani N. Naligama, Anupama P. Halmillawewa

**Affiliations:** a Department of Microbiology, Faculty of Science, University of Kelaniya, Kelaniya, Sri Lanka; USDA San Joaquin Valley Agricultural Sciences Center

**Keywords:** *Pectobacterium carotovorum*, carrot soft rot, bacteriophage, biocontrol, *Certrevirus*, bacteriophage genomics

## Abstract

Pectobacterium carotovorum is an economically important phytopathogen and has been identified as the major causative agent of bacterial soft rot in carrots. Control of this phytopathogen is vital to minimizing carrot harvest losses. As fully efficient control measures to successfully avoid the disease are unavailable, the phage-mediated biocontrol of the pathogen has recently gained scientific attention. In this study, we present a comprehensive characterization of the *P. carotovorum* phage vB_PcaM_P7_Pc (abbreviated as P7_Pc) that was isolated from infected carrot samples with characteristic soft rot symptoms, which were obtained from storage facilities at market places in Gampaha District, Sri Lanka. P7_Pc is a myovirus, and it exhibits growth characteristics of an exclusively lytic life cycle. It showed visible lysis against four of the tested P. carotovorum strains and one Pectobacterium aroidearum strain. This phage also showed a longer latent period (125 min) than other related phages; however, this did not affect its high phage titter (>10^10^ PFU/mL). The final assembled genome of P7_Pc is 147,299 bp in length with a G+C content of 50.34%. Of the 298 predicted open reading frames (ORFs) of the genome of P7_Pc, putative functions were assigned to 53 ORFs. Seven tRNA-coding genes were predicted in the genome, while the genome lacked any major genes coding for lysogeny-related products, confirming its virulent nature. The P7_Pc genome shares 96.12% and 95.74% average nucleotide identities with *Cronobacter* phages CR8 and PBES02, respectively. Phylogenetic and phylogenomic analyses of the genome revealed that P7_Pc clusters well within the clade with the members representing the genus *Certrevirus*. Currently, there are only 4 characterized *Pectobacterium* phages (*P. atrosepticum* phages phiTE and CB7 and *Pectobacterium* phages DU_PP_I and DU_PP_IV) that are classified under the genus, making the phage P7_Pc the first reported member of the genus isolated using the host bacterium *P. carotovorum.* The results of this study provide a detailed characterization of the phage P7_Pc, enabling its careful classification into the genus *Certrevirus*. The knowledge gathered on the phage based on the shared biology of the genus will further aid in the future selection of phage P7_Pc as a biocontrol agent.

**IMPORTANCE** Bacterial soft rot disease, caused by *Pectobacterium* spp., can lead to significant losses in carrot yields. As current control measures involving the use of chemicals or antibiotics are not recommended in many countries, bacteriophage-mediated biocontrol strategies are being explored for the successful control of these phytopathogens. The successful implementation of such biocontrol strategies relies heavily upon the proper understanding of the growth characteristics and genomic properties of the phage. Further, the selection of taxonomically different phages for the formulation of phage cocktails in biocontrol applications is critical to combat potential bacterial resistance development. This study was conducted to carefully characterize and resolve the phylogenetic placement of the *P. carotovorum* phage vB_PcaM_P7_Pc by using its biological and genomic properties. Phage P7_Pc has a myovirus morphotype with an exclusively lytic life cycle, and the absence of genes related to lysogeny, toxin production, and antibiotic resistance in its genome confirmed its suitability to be used in environmental applications. Furthermore, P7_Pc is classified under the genus *Certrevirus*, making it the first reported phage of the genus of the host species, *P. carotovorum*.

## INTRODUCTION

Bacterial soft rot is a globally widespread disease, often caused by the economically important pathogenic bacterial group known as soft rot *Pectobacteriaceae* (SRP; formerly known as soft rot *Enterobacteriaceae*). The SRP includes the two genera *Pectobacterium* and *Dickeya* (1, 2) and is typically known to produce extracellular plant cell wall-degrading enzymes (PCWDEs), such as cellulase, pectinase, and polygalacturonase, during infections, which can cause plant tissue maceration that leads to pre- and postharvest losses in a wide range of economically important crops (3–5), including carrots, potatoes, cabbages, tomatoes, and lettuce (6). The genus *Pectobacterium*, which was first established in 1945, shows great genetic heterogeneity with several species and subspecies (7–9). Earlier, the *P. carotovorum* species was subdivided into several subspecies and proposed subspecies, including *P. carotovorum* subsp. *carotovorum*, *P. carotovorum* subsp. *atrosepticum*, *P. carotovorum* subsp. *betavasculorum*, *P. carotovorum* subsp. *odoriferum*, and *P. carotovorum* subsp. *wasabiae.* More recently, *P. carotovorum*, *P. atrosepticum*, *P. betavasculorum*, *P. wasabiae*, and *P. odoriferum* were elevated to species level (8–10).

*P. carotovorum* (formerly classified as Pectobacterium carotovorum subsp. *carotovorum*), the major causative agent of soft rot in carrots, is identified as one of the most economically significant species of the group, based on its ability to cause a serious impact on carrot harvests under relatively high-humidity and high-temperature conditions of temperate regions (11, 12). Harvest losses in carrots induced by soft rot disease-causing pathogenic bacteria must be decreased to meet the rising global food demands connected to the expansion of the human population. Although the phytosanitary procedures and removal of damaged tubers and plants to prevent the spread of the disease are normally being practiced by farmers to avoid harvest and postharvest losses, these methods are not fully efficient (13, 14).

Bacteriophages, or bacterial viruses, have attracted researchers' interest since their discovery (15), as they provide an environmentally acceptable, attractive, and safe method to control phytopathogens. Bacteriophages infect their respective bacterial hosts to complete their life cycle (15). Their ability to be utilized as biocontrolling agents against soft rot bacteria has been proposed and demonstrated via analysis of the phages of numerous well-studied SRP members. Studies with tubers of potatoes employing phages of SRP have shown that *P. carotovorum*, *P. atrosepticum*, *P. parmentieri*, and Dickeya solani, which induce soft rot in potatoes, could be prevented via phage biocontrol (13, 14, 16, 17). Furthermore, phage vB_DsoM_LIMEstone1 has been used against *D. solani* in a field trial, where it was demonstrated that phage therapy for blackleg in potatoes led to less severe disease in the field and higher potato harvests (17, 18). However, *in silico* analyses of whole genomes of bacteriophages have revealed some intriguing genotypic properties of phages, such as the existence of antibiotic resistance genes, lysogeny-related genes, and toxin-producing genes (15), which may have the potential to negatively contribute when applying them as biocontrolling agents. Such attributes should be taken into careful consideration when selecting potential phage candidates for biocontrol in order to avert any undesirable environmental and ecological consequences (15). Owing to the recent advances in the next-generation sequencing technologies and comparatively lower costs associated with sequencing, the number of phage genomes released into the public databases has increased considerably (19, 20). As the amount of genomic data has expanded, researchers have gained a better grasp of the evolutionary relationships between phages. Although initially phages were classified based on their shape, physicochemical characteristics, and nucleic acid content (21), as technology has progressed, nucleotide and protein homologies have become prominent methods for determining phage phylogenetic relationships.

Many of the reported phages which infect SRP pathogens, such as *P. carotovorum* and *P. atrosepticum*, are classified under the class *Caudoviricetes* (22–24). Phage Arno 160 and phage PP2 are podoviruses that infect *P. carotovorum*, and they belong to the genus *Wanjuvirus* of subfamily *Melnykvirinae* (24, 25). *Pectobacterium* phage My1 was the first reported phage with the siphovirus morphotype that infects *P. carotovorum*, and it belongs to the subfamily *Mccorquodalevirinae* and the genus *Myunavirus* (22). Phage PM1 of genus *Suwonvirus* (subfamily *Cleopatravirinae*) is a myovirus that infects *P. carotovorum* (26). However, only a few phages of *P. carotovorum* with the myovirus morphotype have been studied and reported so far (26, 27). Even though all these *P. carotovorum* phages are classified under the class *Caudoviricetes*, they exhibit great phylogenomic diversity and hence are placed in different subfamilies according to the classification of the International Committee on Taxonomy of Viruses (ICTV) (https://ictv.global/taxonomy).

According to the recent update in ICTV taxonomy release 37 in 2021, the genus *Certrevirus* under the subfamily of *Vequintavirinae* of class *Caudoviricetes* consists of four *Cronobacter* phages (CR3, CR8, CR9, and PBES 02) and one *P. atrosepticum* phage (phiTE). Furthermore, phylogenomic studies and proteome analysis of *P. atrosepticum* phage vB_PatM_CB7 has exhibited a close relationship with these *Cronobacter* phages as well as with the phage phiTE of the genus *Certrevirus*, positioning it within the same genus as well (23). Based on our knowledge, the genus *Certrevirus*, being the first established genus identified and with members infecting *P. atrosepticum* (23), has no reported candidate phage of the host *P. carotovorum*. In this study, we report the general and genomic characterization of a newly isolated *P. carotovorum* phage, vB_PcaM_P7_Pc (hereon, abbreviated as P7_Pc), which fits into the genus *Certrevirus* based on phylogenetic and phylogenomic studies. The morphological and genomic characteristics of phage P7_Pc show similarities to the reported *Certrevirus* phages, providing further understanding of the genus. Additionally, phylogenomic awareness and biodata of the *P. carotovorum* phage P7_Pc gathered through this study can greatly aid in deciding its fitness as a biocontrol agent against the phytopathogen.

## RESULTS AND DISCUSSION

### Isolation, host range, and general characteristics.

Phage P7_Pc was isolated from characteristic diseased carrot tubers collected from commercial outlets in Gampaha District, Sri Lanka, in 2019 by using *P*. *carotovorum* strain DSM 30168 as the host. After the isolation and purification through three consecutive single-plaque purifications, the phage isolate was subjected to further characterization. Phage P7_Pc produced clear plaques ≈0.5 to 1 mm in diameter on 0.7% nutrient agar overlay under laboratory conditions. The host range of P7_Pc was examined against 11 bacterial strains from 6 different species of the genus *Pectobacterium*. *P. carotovorum*^T^ (DSM 30168), *P. atrosepticum*^T^ (DSM 18077), *P. odoriferum*^T^ (DSM 22556), and *P. betavasculorum*^T^ (DSM 18076), as well as seven previously isolated and characterized strains of *P. carotovorum* (C1B5, C2B6, C2B7, and C2B8), *P. aroidearum* (C1B3 and C1B4), and *P. polaris* (C3B9) (28) were used in the host range analysis. The phage was capable of forming clear plaques against not only its host strain, *P. carotovorum* (DSM 30168), but also with several other *P. carotovorum* strains, C2B6, C2B7, and C2B8 (Table 1). P7_Pc formed opaque plaques with *P. aroidearum* C1B3, and it failed to form visible plaques on other species of the genus used in this study. The results of the host range studies suggested that P7_Pc has a narrow host range, where its lytic capacities are limited more or less to a single species of the genus. The subfamily *Vequintavirinae* SRP phage CB7 also exhibited a limited host range, where it was only capable of infecting its host, *P. atrosepticum* DSM 30186, and four other strains of the same species of the 31 bacterial strains belonging to five different species against which it was tested (23).

**TABLE 1:** Host range of *Pectobacterium* phage P7_Pc on 11 strains of *Pectobacterium* as determined by spot assays on agar overlays

Bacterial host		Sensitivity^*a*^
*Pectobacterium* species	Strain	
*P. carotovarum*	DSM 30168^*b*^	+
	C1B5	−
	C2B6	+
	C2B7	+
	C2B8	+
*P. odoriferum*	DSM 22556^*b*^	−
*P. atrosepticum*	DSM 18077^*b*^	−
*P. betavasculorum*	DSM 18076^*b*^	−
*P. aroidearum*	C1B3	+
	C1B4	−
*P. polaris*	C3B9	−

a +, lysis; −, no lysis.

b Type strain for the species.

**FIG 1:**
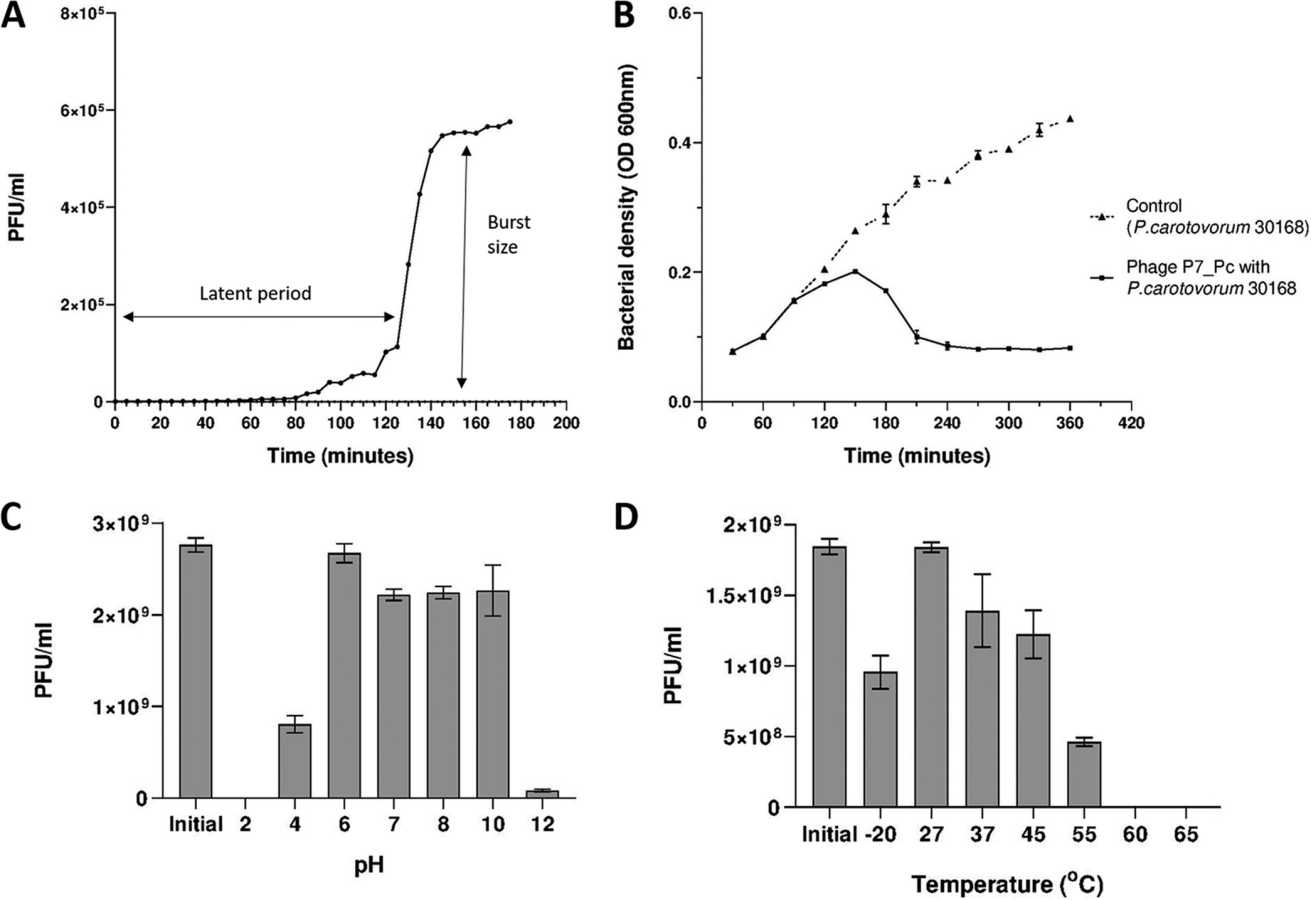
Growth characteristics of *Pectobacterium* phage P7_Pc. (A) One-step growth curve of P7_Pc with its host, *P. carotovorum* DSM 30168, at 28°C. (B) Lytic curve of *Pectobacterium* phage P7_Pc with its host, *P. carotovorum* DSM 30168, at 28°C in nutrient broth (MOI, ≈ 1). (C and D) Stability of *Pectobacterium* phage P7_Pc after exposure to different pH conditions ranging from 2 to 12 for 24 h (C) and incubation at different temperatures for 1 h (D). Error bars represent the standard errors of means (*n* = 3).

The one-step growth curve of phage P7_Pc was used to determine the latent period and burst size of the phage. The results showed that phage P7_Pc has a latent period and a burst size of 125 min and 254 PFU/cell, respectively (Fig. 1A). *Pectobacterium* phage vB_PatM_CB7 of the genus *Certrevirus* has a comparatively smaller burst size (154 PFU/cell) and shorter latent period (55 min). However, another phylogenetic neighbor of P7_Pc, CR3- like phage *Cronobacter* phage PBES 02, exhibits an approximately similar burst size (250 PFU/cell) with a much shorter latent period of 30 min (23). Bacterial growth and factors affecting bacterial growth rates, such as growth medium and other growth conditions, can majorly influence phage growth characteristics such as latent period, burst size, and rate of adsorption. Further, these phage growth parameters may vary based on the different host bacterial species used in an experiment (29).

The infection curve of phage P7_Pc was used to analyze the lytic ability of the phage by determining the optical density at 600 nm (OD_600_) of the infected host cell culture after different time intervals (Fig. 1B). The test was performed under standard growth conditions using nutrient broth medium. The absorbance of the bacterial growth was compared with the OD_600_ of bacterial culture infected with P7_Pc. According to the resulting graph, onset of lysis by P7_Pc was observed between 90 and 120 min after the inoculation, where a drop in the OD was detected compared to the control. At 180 min after the phage inoculation, a steady drop in the OD_600_, which was due to the lysis of bacterial cells by the phage, was observed until it reached a steady state. The average growth reduction of the host bacterium during the P7_Pc infection was determined to be ~81% based on the OD_600_ values of the control and phage-infected bacterial cultures.

The stability of phage P7_Pc was also tested under different temperature and pH conditions. The phage’s pH stability was examined over a 24-h period, using suspension medium adjusted to different pH levels. After the incubation and plating, P7_Pc was stable at the pH range of 4 to 10 (Fig. 1C). Phage P7_Pc was capable of maintaining its viability when stored at −20°C, 27°C, 37°C, 45°C, and 55°C for a period of 1 h (Fig. 1D). However, except at 27°C, storing at −20°C, 37°C, 45°C, and 55°C resulted in a reduction of the phage titer, whereas the percentage decline of the phage titer was ~75% when stored at 55°C. Moreover, no viable phages were detected after the incubation at temperatures higher than 60°C, indicating its instability at higher storage temperatures. Although phage CB7 was also reported to have similar stability ranges (stable between pH 5 and 11 and temperatures of −18°C and 55°C) as P7_Pc, CB7 had a lower percentages of decline of phage titer at higher and lower tolerable temperatures, as well as at pH 4 (23).

### Transmission electron microscopy.

**FIG 2:**
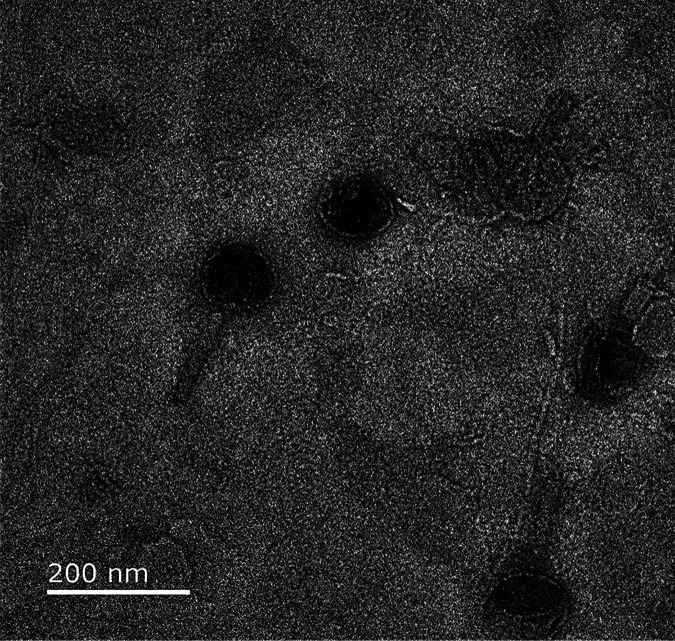
Transmission electron micrographs of *Pectobacterium* phage vB_PcaM_P7_Pc stained with 1% (wt/vol) ammonium molybdate and imaged at 120 kV. Scale bar, 200 nm.

Transmission electron microscopy (TEM) analysis of phage P7_Pc revealed that the phage is a myovirus (Fig. 2) with an icosahedral head and a contractile tail that belongs to the A1 morphotype (30). The head diameter of the phage was estimated to be 89.7 ± 5.5 nm (*n *= 8; mean ± standard error of the mean), and the tail length and width were determined to be 120.3 ± 4.4 nm (*n *= 6) and 16.4 ± 2.8 nm (*n *= 6), respectively. Reportedly, *P. atrosepticum* phage CB7 (head diameter of ~84 nm, tail length of ~123 nm, and tail width of ~20 nm) and *Cronobacter* phage PBES 02 (head diameter of ~90 nm and tail length of ~130 nm) of the genus *Certrevirus* exhibit dimensions similar to those of P7_Pc (23, 31).

### General genome characteristics of P7_Pc.

**FIG 3:**
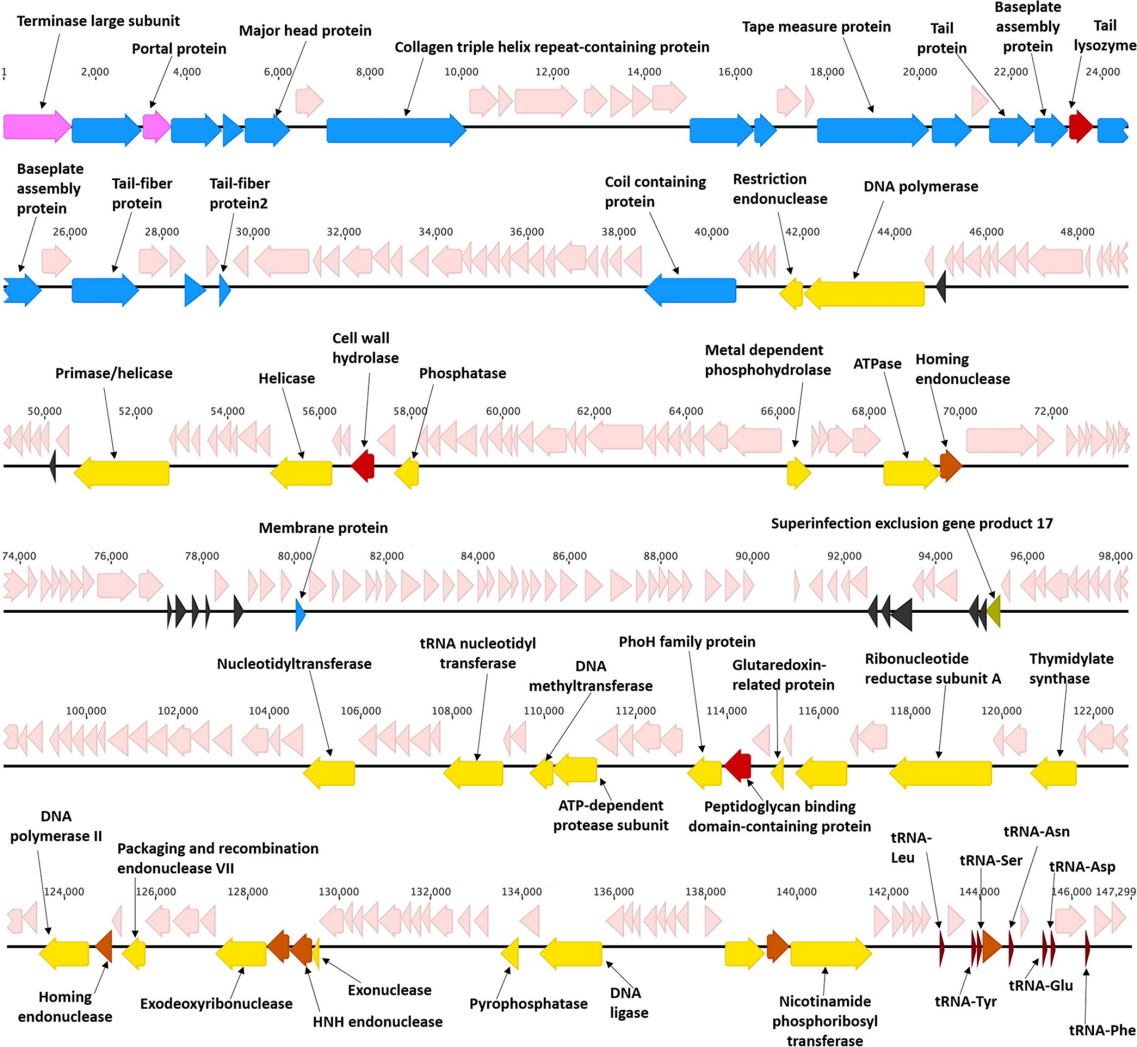
Graphical interpretation of the organization of the 147,299-bp genome of *Pectobacterium* phage P7_Pc. Putative ORFs are represented by arrows and labeled with a predicted function. Nucleic acid metabolism, replication, and transcription genes are colored in yellow; structural genes are in blue; and lysis genes are in red. tRNA genes are in dark red. Hypothetical protein genes are colored in light pink. Putative ORFs are colored in black. The image was created using Geneious (https://www.geneious.com).

The phage lysate was subjected to a polyethylene glycol (PEG) precipitation to increase the phage titer, and thereby the final yield of DNA, before sequencing it using the Illumina platform. The *de novo* assembly of the total number of Illumina MiSeq paired-end reads of 211,392 resulted in a single contig of 147,299 bp (*N*_50_, 147,299). The self-mapping of the assembled genome with raw data reads showed that 99.96% of the total reads were mapping against the assembled read with 100% mapping coverage and an average 309.08 mapping depth. The G+C content of the assembled genome of P7_Pc was 50.34%, which was more or less within the range of its host species, *P. carotovorum* (50 to 52%) (32), suggesting its possible host specificity. The ends of the phylogenetic neighbors of phage P7_Pc, phages CB7 and PhiTE, were identified as circularly permuted using an experimental restriction enzyme digestion analyses (23, 33). Based on the nature of the ends of its relative phages, the P7_Pc genome map was drawn considering the gene coding for the terminase large subunit as the start (Fig. 3).

Based on the annotation results, 298 open reading frames (ORFs) were predicted in the genome of phage P7_Pc (see Table S1 in the supplemental material). Protein sequence similarity searches, protein classification into families and domains, and lipoprotein and transmembrane analyses enabled in assigning putative functions to 53 of 298 predicted ORFs (~17.8%). The remaining ORFs were categorized as hypothetical proteins (208 ORFs; ~69.8%), putative ORFs (11 ORFs; ~3.7%), or transmembrane proteins (26 ORFs; ~8.7%). The possibly exclusive lytic life cycle of the phage was hinted at by the absence of any predicted ORF encoding integrase, excisionase, or repressor protein. Although the genome of P7_Pc was devoid of putative genes coding for major functions associated with lysogenic lifestyle, an ORF predicted to code for superinfection exclusion gene product 17 (P7_194) was identified, and this should be further investigated. However, a domain search of the particular gene using InterProScan showed that the superinfection exclusion gene product 17 could also be found in other bacteriophages, such as phage SPP1. In the lytic Bacillus subtilis siphophage SPP1, this gene product was discovered to function as a tail completion protein that was present between the interface of the head-to-tail connector and the tail of bacteriophage SPP1 with an alpha/beta-fold (34). Although further supporting studies are required to establish the exact function of this gene product in the P7_Pc life cycle, based on the growth characteristics of the phage, it is predicted that the superinfection exclusion gene product 17 might have a similar structure-related function in the P7_Pc genome, as it is most likely to follow an exclusively lytic life cycle.

### tRNA genes and codon usage of the P7_Pc genome.

Both tRNAScan-SE and ARAGORN identified 7 putative tRNA genes in the P7_Pc genome with anticodons corresponding to 7 amino acids: leucine, tyrosine, serine, aspartic acid, glutamic acid, asparagine, and phenylalanine. The most common translation-associated genes found in phage genomes have been identified as putative tRNAs (35). Although usually phages totally depend upon host tRNA genes for translation, sometimes multiple tRNAs can be found in some phage genomes. Among the currently identified members of the genus *Certrivirus*, a great variation in the number of tRNA genes present in the genome is observed, with the *Cronobacter* phage CR3 (18 tRNA genes) and *Pectobacterium* phage vB_PatM_CB7 (1 tRNA gene) harboring the highest and lowest numbers of tRNA genes, respectively (23, 36). Two other *Pectobacterium* phages, DU_PP_I (MF979560) and DU_PP_IV (MF979563), which are attributed to the same genus according to their GenBank records, have 8 tRNA genes in each of their genomes. A higher number of tRNA genes are observed in *Certrivirus* phages infecting *Cronobacter* (14 to 18), while the numbers of tRNA genes vary from 1 to 8 among the *Pectobacterium* phages of the genus (23).

The distribution of tRNAs in phage genomes is frequently linked to phage codon usage bias, which shows that certain codons are utilized more frequently than other synonymous codons during gene translation (35). The typically small genome capacities of phages compel them to heavily rely on host cellular apparatus for various functions of their life cycle, including protein synthesis. Although, the ideal strategy for a phage would be to entirely mimic the codon biasness and tRNA usage of its host, this may not be feasible in all instances, especially due to the slight differences in the G+C composition of the genomes of the phage and its bacterial host (35). Phage genomes are known to be marginally A+T richer than their host genomes; hence, they may need to recruit their own tRNA to atone the gap. So, the occurrence of tRNA genes in phage genomes that correspond to codons that are commonly utilized by the phage, but rarely used by the host bacteria, is suggested as a method used by phages to compensate this translational difference (35). Additionally, a higher number of tRNA genes and higher codon bias were observed among virulent phages in terms of the efficient complementation of their virulent life cycle, which requires faster growth rates and translation machineries to increase their competitive fitness (35). To further understand the presence tRNA genes in the P7_Pc genome, we compared the codon usage frequency of phage P7_Pc with that of its bacterial host, *P. carotovorum* DSM 30168 (Table 2).

**TABLE 2:** Codon usage frequencies for phage P7_Pc and bacterial host *P. carotovorum* DSM 30168 for amino acids leucine, tyrosine, serine, aspartic acid, glutamic acid, asparagine, and phenylalanine

Amino acid	Codon^*a*^	Frequency of codon usage (‰)	
		Phage P7_pc	*P. carotovorum* 30168
Leucine	TTG*	13.41624	15.3344764
	TTA	5.103519	10.8134623
	CTG	33.56288	57.215719
	CTA	3.075483	5.70949064
	CTT	12.92594	9.19049685
	CTC	10.34075	10.855674
Tyrosine	TAT	15.3997	16.4174238
	TAC*	22.68726	12.162052
Serine	AGT	6.373827	7.62502656
	AGC	9.092732	18.9275619
	TCG	4.657797	10.0303633
	TCA	6.886408	7.73710579
	TCT	13.72824	8.9088432
	TCC*	11.43277	9.88407805
Aspartic acid	GAT	29.39538	34.0524356
	GAC*	35.54634	18.9770514
Glutamic acid	GAG	35.54634	18.8016547
	GAA*	35.16748	36.6964865
Asparagine	AAT	13.90653	17.5607775
	AAC*	28.32564	20.2754238
Phenylalanine	TTT	13.88425	19.819829
	TTC*	27.36734	17.9989054

a Codons with an asterisk corresponds to the relevant anticodon used by the tRNAs present in the P7_Pc genome.

For the amino acids tyrosine (TAC), aspartic acid (GAC), asparagine (AAC), and phenylalanine (TTC), phage P7_Pc had notably higher codon usage frequencies than its host bacterium, *P. carotovorum* 30168. However, codons TTG, TCC, and GAA, corresponding to amino acids leucine, serine, and glutamic acid, respectively, were not recognized as the highest-used codons for the relevant amino acids in the P7_Pc genome and shared more or less similar codon usage frequencies with the host bacterium.

### DNA packaging and structure-related genes.

In most double-stranded DNA-bearing tailed phages, the DNA packaging motor follows a similar structure, with two neighboring genes coding for two nonstructural components, the big and small terminase subunits (37). However, within the P7_Pc genome, only one putative phage terminase large subunit gene (P7_01) could be clearly identified as a component of the phage DNA packing mechanism, based on functional predictions of genes. In the majority of tailed phages, a gene encoding a portal protein can be found near terminase genes. Double-stranded DNA phages commonly bundle their DNA into a prohead or procapsid, which requires three distinct procapsid proteins to assemble: a coat protein, a scaffolding protein, and a portal protein (37). Genes for a portal protein (P7_03) and two adjacent structural proteins (P7_02, 04) were located near the gene coding for the terminase large subunit, which might play a role in the formation of procapsids and DNA packaging.

In total, 16 ORFs were predicted to be involved in the cluster of virion morphogenesis of P7_Pc. This included 5 proteins involved in the head morphogenesis (P7_02, P7_04, P7_16, P7_17, and P7_21), a portal protein (P7_03), a head stabilization and decoration protein (P7_05), and a putative major head protein (P7_06). Genes that were ascribed for the functions linked to tail morphogenesis included a putative collagen triple helix repeat-containing protein (P7_08), a putative tape measure protein (P7_20), a putative tail protein (P7_23), two putative baseplate assembly proteins (P7_24, and P7_26), a putative tail fiber protein (P7_28), a putative membrane protein (P7_31), and a putative tail fiber protein 2 (P7_33). In addition, genes for two phage tail proteins, a coil-containing protein (P7_55) and a membrane protein (P7_152), were discovered distant from the well-identified tail gene region of the P7_Pc genome. According to BLASTP searches, all the structure-related genes identified in the genome of P7_Pc shared similarities with structural proteins of the members of the genus *Certrevirus*.

### Transcription and terminators.

None of the predicted ORFs of the P7_Pc genome were identified to code for putative RNA polymerases. This aligns with the lifestyle of similar phages, implying its total dependency on host-encoded RNA polymerase for its transcription. Further, 16 rho-independent terminators were recognized in the P7_Pc genome, while none of the ORFs was identified to be coding for any putative transcriptional factors.

### DNA replication, methylation, and nucleotide metabolism.

The P7_Pc genome contained putative protein-coding genes that are required in DNA replication, such as DNA polymerase (P7_61 and P7_252), DNA helicase (P7_92), DNA primase (P7_83), and DNA ligase (P7_276). Furthermore, a putative packaging and recombination endonuclease VII (P7_255), which is required in DNA mismatch repair and involved in DNA packaging, was detected in the genome (38). Methylation is a key natural process of chemical DNA modification (39), and DNA methyltransferases are responsible for methylating the genomes, which is important in escaping the host defense mechanisms employed via restriction endonucleases (40). A putative DNA methyltransferase-coding gene (P7_230) was identified in the P7_Pc genome, and based on the InterProScan search, P7_230 is likely to code for a putative N-6 adenine-specific DNA methylase.

The P7_Pc genome also contains genes encoding proteins related to nucleotide metabolism, such as ribonucleotide reductase (RNR) subunits A (P7_244) and B (P7_241), which play key roles in nucleotide biosynthesis by transforming ribonucleotides into deoxyribonucleotides (41, 42). The transformation can possibly be assisted by glutaredoxin by allowing the regeneration of RNR (P7_239) (43). In addition, the synthesis of deoxythymidine monophosphate (dTMP) from dUMP is supported by a thymidylate synthase (P7_247) (44). The P7_Pcc genome contains two metabolic genes: ribose-phosphate pyrophosphokinase (*prs*; P7_285) and nicotinamide phosphoribosyl transferase (*pncB*; P7_287), which indicate the pyridine nucleotide salvage (45). The phage carries a tRNA nucleotidyl transferase gene (P7_227) for the normal cell growth and reparation of tRNA molecules with a defective 3′-terminal sequence (46, 47).

Homing endonucleases are a type of mobile genetic element that codes for an endonuclease, which facilitates the lateral transfer of its own reading frame (48, 49). Homing endonucleases are usually known to be associated with self-splicing elements, such as inteins and introns, within genomes. The genome of phage CB7 has 21 ORFs encoding HNHs, and these represent 7% of its total genome (23). Among 21 of these HNHs, 16 HNH-coding genes were present as freestanding forms, while 5 HNH-coding genes were identified to be associated with introns. Six homing endonuclease genes were identified in the P7_Pc genome (P7_124, P7_253, P7_260, P7_261, P7_286, and P7_294) and, unlike with the phage CB7, all these were freestanding homing endonucleases. Further, no inteins were identified in the P7_Pc genome.

### Cell wall-degrading enzymes and cell lysis proteins.

Phages have the ability to penetrate the cell wall of the bacterial host, which is mainly made up of peptidoglycan, during the initial stage of phage infection. At the end of its lytic cycle, the host cell should lyse in order to release the progeny phages (50). A putative cell wall hydrolase-coding gene with a signal peptide sequence (P7_95) was predicted in the P7_Pc genome. The gene showed similarities to putative cell wall hydrolase genes of phages CR3 (CR3_87), CR8 (CR8_90), phiTE (phiTE_33), and CB7 (CB7_83). It was reported that homologs of this cell wall hydrolase resembling *Sle*B were identified among the studied members of all genera of the *Vequintavirinae* subfamily (23). A putative tail lysozyme (P7_25), an ATP-dependent protease subunit (P7_231), and a peptidoglycan-binding lysozyme-like domain-containing protein (P7_237) which might have the potential to degrade the host cell wall during an infection were also identified in the P7_Pc genome. However, in P7_Pc a classic lysis cassette could not be identified, where all the lysis genes would be present closer to each other. This has also been seen in other members of the genus *Certrevirus*; especially as they are larger phages, the lysis genes will be distributed throughout the genome (23).

### Phylogenetic and phylogenomic analyses.

**FIG 4:**
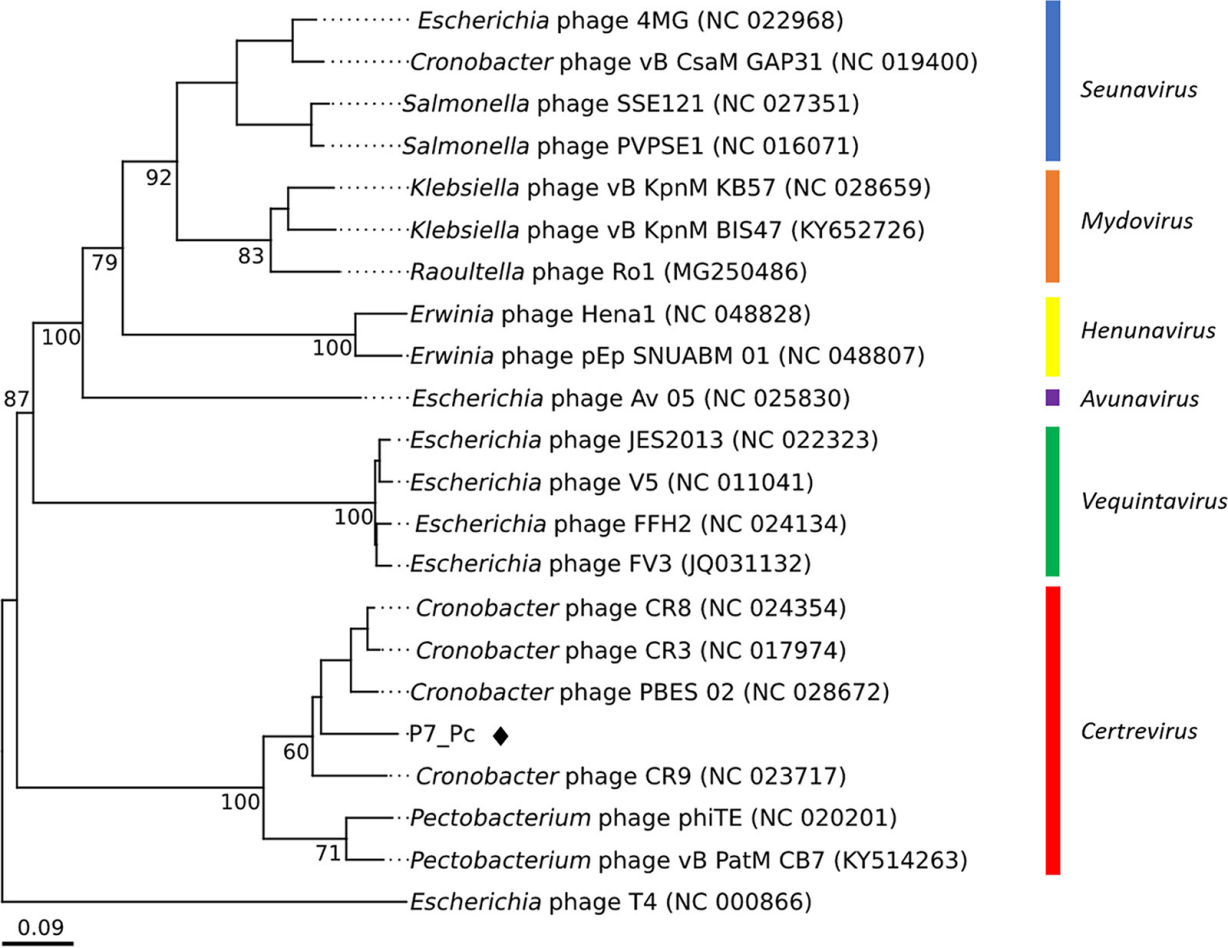
VICTOR-generated nucleotide phylogenomic GBDP tree of *Pectobacterium* phage P7_Pc (♦) and 21 members of the *Vequnitavirinae* subfamily inferred using the formula D0 and yielding average support of 60%. The numbers below branches are GBDP pseudobootstrap support values from 100 replications. Members of the genera *Seunavirus*, *Mydovirus*, *Henunavirus*, *Avunavirus*, *Vequintavirus*, and *Certrevirus* are illustrated. Escherichia virus T4 was used as the outgroup. Scale bar, 0.09 nucleotide substitutions per character.

**FIG 5:**
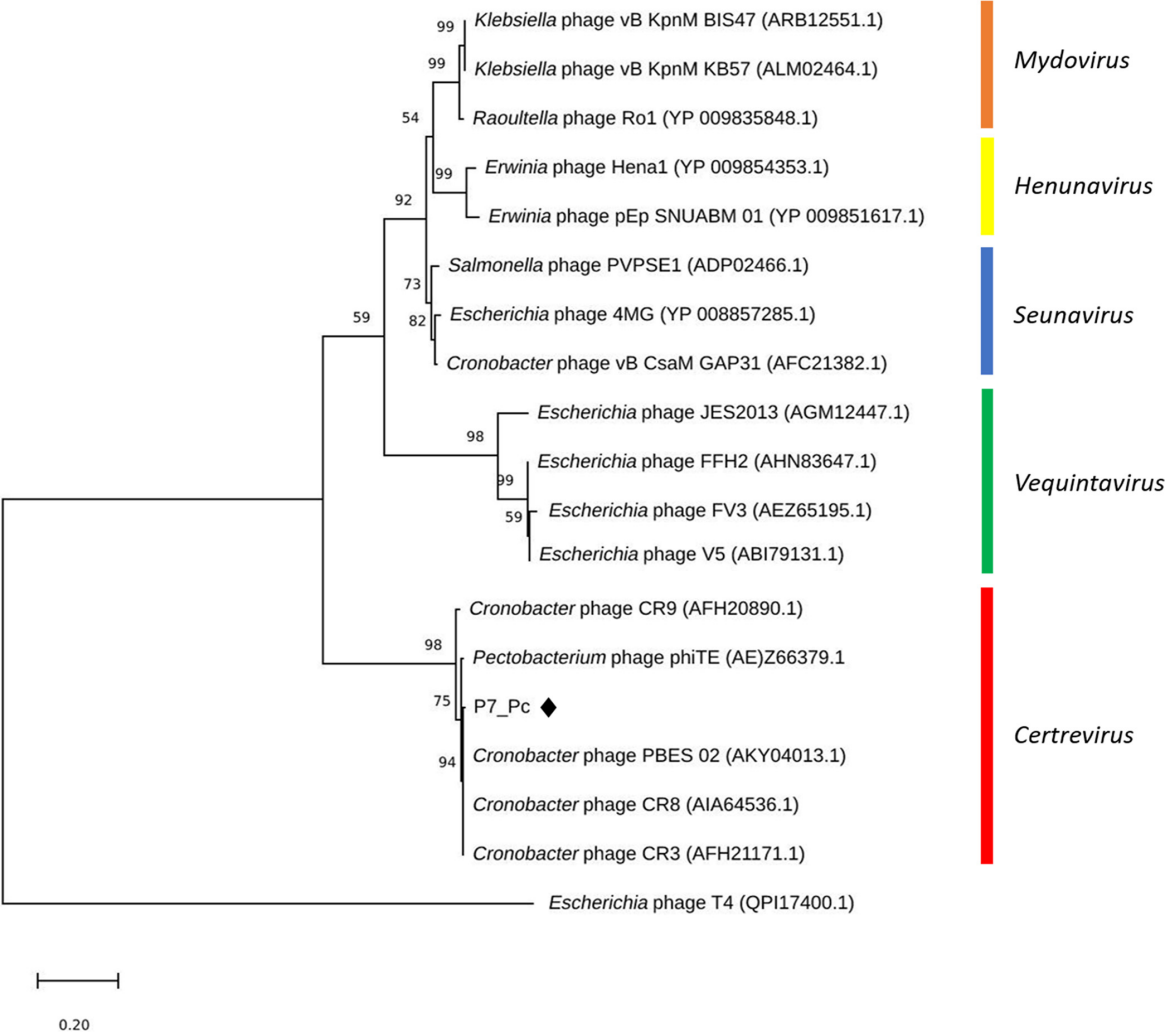
Phylogenetic analyses of amino acid sequences of the major capsid protein gene of phage P7_Pc (♦) and 17 members of the *Vequnitavirinae* subfamily using maximum likelihood method based on Whelan and Goldman model with frequencies (+F) with 1,000 bootstrap replicates. Members of the genera *Seunavirus*, *Mydovirus*, *Henunavirus*, *Vequintavirus*, and *Certrevirus* are illustrated. Escherichia virus T4 was used as outgroup of the analysis. Scale bar, 0.2 nucleotide substitutions per character.

**FIG 6:**
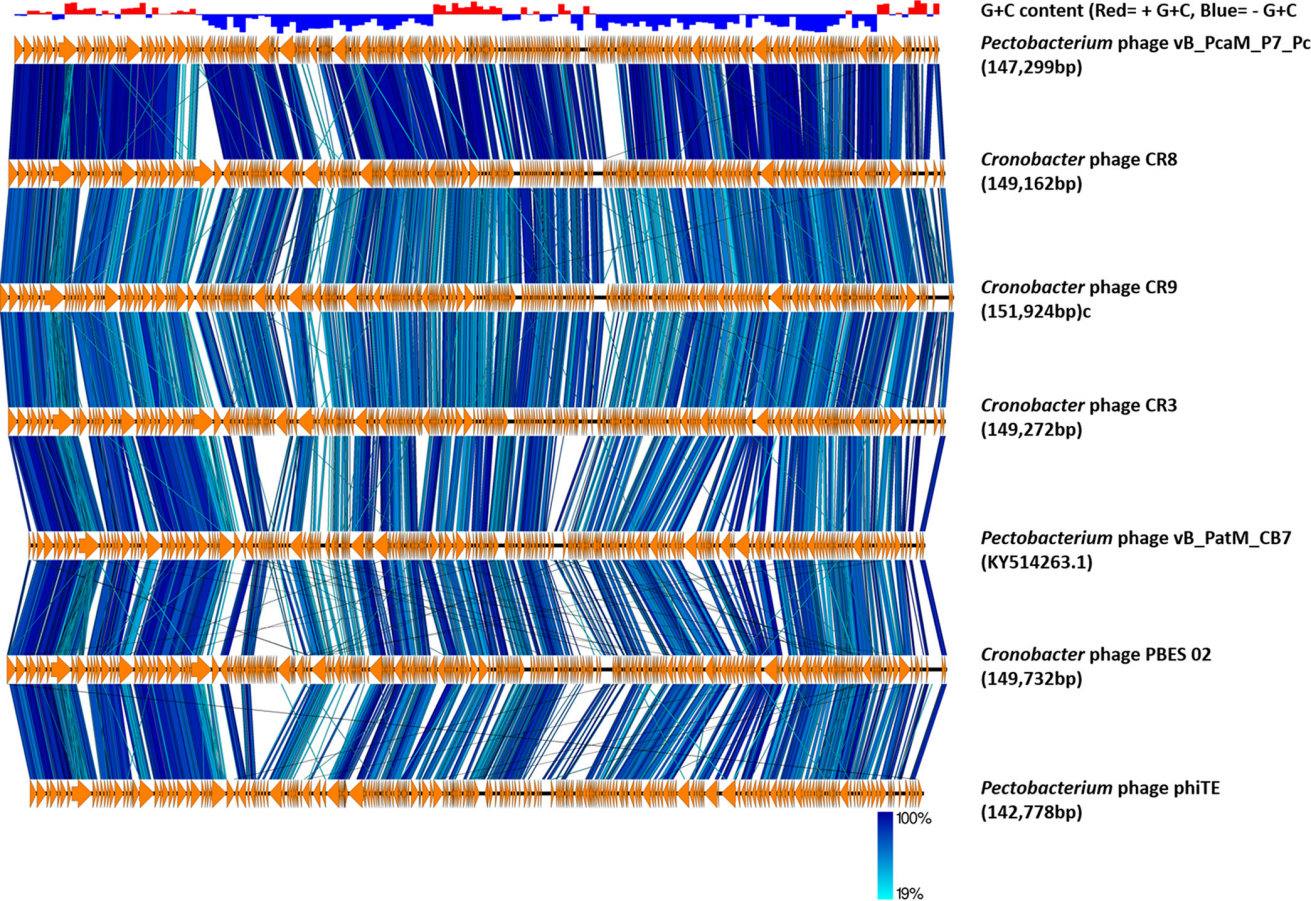
Genome comparison of *Pectobacterium* phage P7_Pc with six other members of the genus *Certrevirus*. The current annotations available in GenBank were used for the comparisons, performed with TBLASTX using EasyFig 2.2.5 (74). Orange arrows show the gene locations and orientations in different phage genomes, and blue lines between the genome maps illustrate the identity level. The phiTE and PBES 02 genomes were rearranged to set the large terminase gene as the first gene.

Creation and examination of a genome-BLAST distance phylogeny (GBDP) phylogram of the P7_Pc genome using VICTOR showed that the phage possesses a notable similarity with phages of the genus *Certrevirus*, as it is well clustered within a clade representing *Certrevirus* (Fig. 4). The phylogenetic tree of major capsid proteins of the members of the subfamily *Vequintavirinae* that was generated using maximum likelihood method further confirmed this observation (Fig. 5), where P7_Pc was placed within the clade containing the representing members of the *Certrevirus* genus. Furthermore, a significant sequence identity was observed during the genome-wide comparison of the P7_Pc genome with five other members of the genus *Certrevirus* employing TBLASTX (Fig. 6) and BLASTN (see Fig. S2 in the supplemental material), illustrating their closer relationship.

According to the recent update in ICTV taxonomy release 37 in 2021 (https://ictv.global/taxonomy), the genus *Certrevirus* under the subfamily of *Vequintavirinae* of class *Caudoviricetes* has 5 members: *Caulobacter* phages CR3, CR8, CR9, and PBES02 and *Pectobacterium* phage PhiTE. Based on the GenBank records, two *Pectobacterium* phages, DU_PP_I and DU_PP_IV, are also classified under the genus (accession numbers MF979560 and MF979563). Furthermore, a recent publication indicated that the *Pectobacterium* phage vB_PatM_CB7 also clustered within the genus *Certrevirus* (23). The genome characteristics of phage P7_Pc demonstrated a notable compatibility with that of the other reported *Certrevirus* members (Table 3). The sizes of the *Certrevirus* phage genomes vary between 142,349 bp (phiTE) and 151,924 bp (CR9) with an average G+C content of 50.43 ± 0.32%. Within the *Certrevirus* genus, *Pectobacterium* phage P7_Pc has the highest reported number of ORFs (298). *Cronobacter* phages of the genus harbor a comparatively higher number of tRNA genes, with 18 tRNA genes in *Cronobacter* phage CR3 being the highest number reported. *Pectobacterium* phages DU_PP_I and DU_PP_IV contain 8 tRNA genes each in their genomes, while only one tRNA gene was detected in phage CB7. Seven tRNA genes were identified in the genome of P7_Pc, which is closer to the number of tRNAs present in DU_PP_I and DU_PP_IV phages. A pairwise comparison of average nucleotide identities (ANIs) between P7_Pc and other phages of the genus revealed that the *Cronobacter* phage CR8 shared the highest nucleotide identity (96.1%) with phage P7_Pc, while the lowest identity was noted between P7_Pc and *Cronobacter* phage CR9.

**TABLE 3:** Comparison of the P7_Pc genome with genomes of five phages currently forming the genus *Certrevirus*, according to the ICTV 2021 report, and three other *Pectobacterium* phages, vB_PatM_CB7, DU_PP_I, and DU_PP_IV

Phage name (GenBank accession no)	Genome size (bp)	G+C content (%)	No. of ORFs	No of tRNAs	Identity^*a*^ (%)
*Cronobacter* phage CR3 (NC_017974.1)	149,273	50.9	265	18	94.93
*Cronobacter* phage CR8 (NC_024354.1)	149,162	50.8	269	17	96.12
*Cronobacter* phage PBES 02 (NC_028672.1)	149,732	50.7	270	14	95.74
*Cronobacter* phage CR9 (NC_023717.1)	151,924	50.6	281	17	82.75
*Pectobactreium* phage phiTE (NC_020201.1)	142,349	50.1	242	2	86.98
*Pectobacterium* phage vB_PatM_CB7 (KY514263.1)	142,778	50.1	253	1	87.67
*Pectobacterium* phage DU_PP_I (MF979560)	144,959	50.1	267	8	93.99
*Pectobacterium* phage DU_PP_IV (MF979563)	145,233	50.3	268	8	94.17
*Pectobactreium* phage vB_PcaM_P7_Pc (ON712643)	147,299	50.3	298	7	100

a Average nucleotide identity (ANI) between genomes compared with that of *Pectobactreium* phage P7_Pc, using an ANI calculator (http://enve-omics.ce.gatech.edu/ani/) (75).

The results of the phylogenomic, phylogenetic, and comparative genomic analyses of the P7_Pc genome reinforced its position within the genus *Certrevirus*. The careful classification of phages into specified genera enables the precise understanding of their shared biology and, more importantly, aids in their selection as biocontrol agents. The simultaneous use of multiple phages or phage mixtures is usually considered under phage-mediated biocontrol strategies. The use of taxonomically diverse phages for the development of phage cocktails is greatly recommended, as such phages have the potential to recognize different host cell receptors during attachment and therefore can reduce the subsequent development of bacterial resistance against phages (51, 52), leading to risk-free and efficient biocontrolling methods.

### Conclusion.

In this study, we have characterized the phage vB_PcaM_P7_Pc isolated using *P. carotovorum* DSM 30168 as the trapping host. The *in silico* analysis of its complete genome, as well as phylogenetic and phlyogenomic comparisons, assisted in classifying the phage P7_Pc taxonomically under the genus *Certrevirus*, making it the first reported *P. carotovorum* phage of the genus.

## MATERIALS AND METHODS

### Isolation of bacteriophages.

For the isolation of phage, 10 g of diseased carrot sample was added into a 25 mL of a log-phase culture of the trapping host grown in nutrient broth. *P. carotovorum* DSM 30168 was used as the trapping host to isolate the phage. After incubating for 24 h at 28°C, culture was allowed to settle, and chloroform-treated aliquots of the supernatant were centrifuged to pellet the bacterial cells and any remaining carrot debris. A 100-μL aliquot of the supernatant was removed into a clean Eppendorf tube and mixed with 300 μL of host bacterial culture. The supernatant with the bacterial host was plated on nutrient agar (NA) using the agar overlay method (53). After incubation at 28°C, individual plaques were picked and purified using three successive single-plaque isolations. The isolated phage in the suspension medium was stored at 4°C and used for further analyses.

### Host range analysis.

The ability of the phage isolate to lyse different bacterial isolates was screened using spot assays as described by Adams (54). A tube containing 5 mL of molten soft NA (0.7% agar [wt/vol]) was inoculated with 300 μL of exponentially growing bacterial host culture (OD_600_, ≈0.3 to 0.4). The tubes were briefly mixed and poured evenly on an NA plate to obtain a lawn of bacterial host culture. After solidification, the surface of the bacterial lawn was spotted with 10 μl of the purified phage suspension. The plates were observed after an overnight incubation at 28°C, either for “lysis” (plaque production) or “no lysis” (no plaque production). The type of lysis observed was reported. Eleven different *Pectobacterium* strains that belonged to 6 different species, namely, *P. carotovorum*, *P. atrosepticum*, *P. odoriferum*, *P. betavasculorum*, *P. aroidearum*, and *P. polaris*, were used in this assay.

### One-step growth analysis.

One-step growth curve analysis of the phage was carried out using previously described methods (23). Briefly, host bacteria were grown to an OD_600_ of 0.3 to 0.35 (approximately 1 × 10^8^ CFU/mL), and 2 to 5 mL of the culture was centrifuged in a microcentrifuge tube to pellet bacterial cells. The pellet was resuspended in 1 mL of the phage lysate to yield an approximate multiplicity of infection (MOI) of 5 × 10^−6^. Then, it was incubated at 28°C for 1 min and centrifuged to pellet the bacteria, and the supernatant was removed to separate bound phage from unbound phage. The bacterial pellet with bound phage was then resuspended in 10 mL of fresh nutrient broth and incubated at 28°C on a shaking incubator (60 to 80 rpm). Aliquots were removed at 5-min intervals to determine the phage titer by the overlay method. Based on the number of PFU per milliliter, the latent period and burst size were determined.

### Lytic activity of P7_Pc.

To determine the lytic activity of P7_Pc, a flask containing 25 mL of nutrient broth was inoculated with the host bacterial culture and incubated overnight with constant aeration at 28°C. The culture (2 mL) was added to 150 mL of fresh nutrient broth and incubated with constant aeration at 28°C (until the culture reached an approximate OD_600_ of 0.2). Then, a 25-mL aliquot of the culture was transferred aseptically to each of the six empty 100-mL sterile flasks. One milliliter of the phage suspension was added to each of the three flasks to obtain an approximate MOI of 1, and the other three were used as controls. Absorbance of the culture (OD_600_) was measured at the initial point, and the flasks were incubated with shaking at 28°C. Samples were withdrawn at 30-min intervals for 6 to 7 h, and the OD_600_ of the samples was recorded to observe the onset of phage lysis.

### Stability of P7_Pc.

The ability of phages to withstand varying environmental conditions is an important characteristic of a potential biocontrol agent. Therefore, the viability of P7_Pc was determined after incubating at different temperatures and pH conditions. Phage aliquots with known titers were added to suspension buffer (50 mM Tris-HCl [pH 7.5], 100 mM NaCl, 8 mM MgSO_4_) and incubated at different temperatures for 1 h. Similarly, the pH of the suspension buffer was adjusted using 1 M HCI or 1 M NaOH to obtain a series of solutions with pH ranging from 2 to 12. Phage aliquots were added to different pH solutions and incubated for 24 h. After the incubation, phage was enumerated using the agar overlay technique with its respective bacterial host. Three independent replicates for each test were used, and the average phage titers after the incubation were calculated in PFU per milliliter.

### Transmission electron microscopy.

Phage lysate produced from plate cultures of phage P7_Pc was used to prepare high-titer phage lysate (>1 × 10^10^ PFU/mL) using PEG precipitation. Forty milliliters of phage lysate was added to an SS-34 Oakridge tube, and 10 mL of 20% PEG 8000 (wt/vol)–2.5 M NaCl was added to the same tube. Next, the tube was incubated on ice for >30 min and centrifuged at 11,000 × *g* for 20 min to spin down the phage. The supernatant was removed using a micropipette, and the centrifugation step was repeated 2 to 3 times to remove any remaining PEG solution. The resulting pellet was resuspended in 1 mL of Sodium chloride-Tris-EDTA buffer (STE) buffer and transferred into a clean microcentrifuge tube. Then, it was centrifuged at 14,000 × *g* for 10 min and the supernatant was collected into another sterile microfuge tube. The phage lysate was stored at 4°C until further analysis.

The TEM grid (a holey carbon-coated Cu grid, 400 mesh size) was placed on 10 μL of the PEG-precipitated phage suspension and held for 5 to 6 min, followed by a staining step using 1% ammonium molybdate (wt/vol) for 30 s. The grids were observed using a JEOL-JEM-2100 high-resolution transmission electron microscope at the Electron Microscopy Facility of Sri Lanka Institute of Nanotechnology at 120 kV to determine the morphological characteristics of P7_Pc.

### DNA extraction, sequencing, and assembly.

The PEG-precipitated and purified phage lysate of phage P7_Pc was used for the DNA extraction. Genomic DNA was extracted using a Norgen phage DNA extraction kit (Norgen Biotek Corp., Canada), according to the manufacturer's specifications and visualized using a 1.2% agarose gel in TBE.

Phage genome sequencing was done at Macrogen, Korea, using the Illumina platform. The sequencing library was prepared by random fragmentation of the DNA sample, followed by 5′ and 3′ adapter ligation. A Nextera XT DNA library preparation kit (96 samples) was used for the library preparation. SPAdes 3.13.0 was used for the *de novo* assembly, and a single contig was generated.

### *In silico* analysis of the phage genome.

The annotation of the phage genome was carried out using structural and functional workflows available at the Center for Phage Technology (CPT) Galaxy platform (https://cpt.tamu.edu/galaxy-pub). The prediction of possible open reading frames (ORFs) was done with GLIMMER 3 (55) and Metadata annotator (56), available in the CPT-Galaxy platform. For functional annotation of the genome, PAP Functional Workflow v2021.01 of CPT-Galaxy was used. The workflow carries out database searches against NCBI’s NT database, CPT’s Canonical Phage database, the Swiss-Prot (curated from UniProt), and the nr database using BLASTP (56–59). Protein families and functions of the predicted proteins were obtained using InterProScan (https://www.ncbi.nlm.nih.gov/pmc/articles/PMC3998142/) (60, 61). Transmembrane domains and lipoprotein cleavage signals were identified using TMHMM (62) and LipoP (60). The genome was scanned for the presence of tRNA genes using tRNAscan-SE (63) and ARAGON (64). Possible Rho-independent terminators were recognized using TransTermHP (65). The genome was screened for the presence of putative genes encoding known toxins, lysogeny- and integration-related functions, virulence factors, and proteins related to antibiotic resistance. Codon frequency analysis of the phage P7_Pc and its host bacterium, *P. carotovorum* DSM 30168, were done using Codon usage statistics available at CPT Galaxy (66). The graphical interpretation of genome P7_Pc was drawn using Geneious R6 (https://www.geneious.com). The annotated genome of P7_Pc can be accessed under GenBank accession number ON712643.

### Comparative genomics.

MEGA11 (67) was used to create the phylogenetic tree based on the amino acid sequences of the gene coding for the major capsid protein of phages within the class *Caudoviricetes*. MUSCLE (68) was used for sequence alignment, and phylograms were constructed using the maximum likelihood method based on the Whelan and Goldman model with freqs(+F) with 1,000 bootstrap replicates.

Phylogenomic analysis of P7_Pc was conducted using VICTOR (the Virus Classification and Tree Building Online Resource), employing the genome-BLAST distance phylogeny (GBDP) technique with pairwise comparisons of the nucleotide sequences under settings recommended for prokaryotic viruses (69). For each of formulae D0, D4, and D6, the obtained intergenomic distances were utilized to construct a balanced minimum evolution tree with branch support, using FASTME with SPR postprocessing (70). A total of 100 pseudobootstrap repetitions were used to infer branch support. FigTree was used to display trees that were rooted at the midpoint (71, 72). The OPTSIL tool (73), the suggested clustering thresholds (69), and an *F* value of 0.5 (fraction of linkages necessary for cluster fusion) were used to estimate taxon boundaries at the species, genus, and family levels.

The linear genomic comparison map of the five members of the genus *Certrevirus* along with the P7_Pc was generated using TBLASTX and BLASTN homology comparisons separately and visualized using EasyFig 2.2.5 (74).

### Data availability.

The annotated genome of phage P7_Pc can be accessed under the GenBank accession number ON712643.
