# Supplementary material for: Pectobacterium carotovorum Phage vB_PcaM_P7_Pc Is a New Member of the Genus Certrevirus
Source: Microbiol Spectr. 2022 Nov 8;10(6):e03126-22. doi: 10.1128/spectrum.03126-22 (PMC9769974; doi:10.1128/spectrum.03126-22)
Supplement: Supplemental file 1 — Supplemental material. Download spectrum.03126-22-s0001.pdf, PDF file, 1.9 MB [file spectrum.03126-22-s0001.pdf]

Supplementary Materials

Table S.1. Annotations of phage P7_Pc genome: Open reading frames and their predicted functions

Product Name	Start	End	Strand	Putative product Length (AA)	Start Codon	Stop Codon	Best BLASTP match/Accession number/E value
P7_001 – Terminase large subunit	1	1488	+	495	ATG	TAA	Putative terminase large subunit [*Cronobacter* phage CR8]/YP_009042238/0.0
P7_002 – Structural protein	1,507	3030	+	507	GTG	TAA	Structural protein [*Pectobacterium* phage vB_PatM_CB7]/ARB11478/0.0
P7_003 – Portal protein	3,052	3675	+	207	TTG	TGA	Portal protein [*Pectobacterium* phage vB_PatM_CB7]/ARB11479.1/1.60282e-136
P7_004 – Structural protein	3,672	4778	+	368	ATG	TAA	Structural protein [*Pectobacterium* phage vB_PatM_CB7]/ARB11480.1/0.0
P7_005 – Head stabilization/ decoration protein	4,796	5245	+	149	ATG	TAA	Head stabilization/decoration protein [*Pectobacterium* phage phiTE]/ YP_007392674.1/6.63906e-99
P7_006 – Putative major head protein	5,265	6263	+	332	ATG	TAA	Putative major head protein [*Pectobacterium* phage vB_PatM_CB7]/ARB11482.1/0.0
P7_007 – Hypothetical protein	6,384	6998	+	204	ATG	TAA	Hypothetical protein HL10_gp007 [Cronobacter phage CR8]/YP_009042244.1/1.58597e-145
P7_008 – Putative collagen triple helix repeat-containing protein	7,059	10127	+	1022	ATG	TAA	Putative collagen triple helix repeat-containing protein [*Pectobacterium* phage DU_PP_I]/ ATS93408.1/ 0.0
P7_009 – Hypothetical protein	10,169	10819	+	216	ATG	TAA	Hypothetical protein CR3_gp009 [*Cronobacter* phage CR3]/YP_006383024.1/2.04298e-158
P7_010 – Hypothetical protein	10,821	11132	+	103	ATG	TAA	Hypothetical protein CR3_gp010 [*Cronobacter* phage CR3]/YP_006383025.1/7.87743e-63
P7_011 – Hypothetical protein	11,176	12546	+	456	ATG	TAA	Hypothetical protein ADU18_0118 [*Cronobacter* phage PBES 02]/YP_009188979.1/0.0
P7_012 – Hypothetical protein	12,676	13203	+	175	ATG	TAA	Hypothetical protein CB7_13 [*Pectobacterium* phage

							vB_PatM_CB7]/ARB11487.1/9.93759e-125
P7_013 – Hypothetical protein	13,266	13727	+	153	GTG	TAA	Hypothetical protein phiTE_219 [*Pectobacterium* phage phiTE]/YP_007392681.1/2.64608e-111
P7_014 – Hypothetical protein	13,738	14178	+	146	ATG	TAA	Hypothetical protein ADU18_0121 [*Cronobacter* phage PBES 02]/YP_009188982.1/9.31019e-107
P7_015 – Hypothetical protein	14,178	14921	+	247	ATG	TAA	Hypothetical protein phiTE_221 [*Pectobacterium* phage phiTE]/YP_007392683.1/1.92181e-148
P7_016 – Putative structural protein	14,981	16402	+	473	ATG	TAA	Putative structural protein 1 [*Cronobacter* phage CR3]/YP_006383031.1/0.0
P7_017 – Structural protein	16,406	16888	+	160	ATG	TAA	Structural protein [*Pectobacterium* phage phiTE]/YP_007392685.1/1.28919e-110
P7_018 – Hypothetical protein	16,906	17436	+	176	ATG	TGA	Hypothetical protein P1A145kb_p017 [*Pectobacterium* phage DU_PP_I]/ATS93417.1/ 1.0573e-100
P7_019 – Hypothetical protein	17,523	17705	+	60	ATG	TAA	Hypothetical protein P1A145kb_p018 [*Pectobacterium* phage DU_PP_I]/ATS93418.1/ 3.48579e-32
P7_020 – Putative tape measure protein	17,770	20211	+	813	ATG	TAA	Putative tape measure protein [*Cronobacter* phage CR9]/YP_009014981.1/0.0
P7_021 – Structural protein	20,282	21145	+	287	ATG	TAA	Structural protein [*Pectobacterium* phage vB_PatM_CB7]/ARB11498.1/0.0
P7_022 – Hypothetical protein	21,148	21522	+	124	TTG	TAA	Hypothetical protein HL10_gp022 [*Cronobacter* phage CR8]/YP_009042259.1/7.71692e-88
P7_023 – Putative tail protein	21,522	22514	+	330	ATG	TAA	Putative tail protein [*Pectobacterium* phage vB_PatM_CB7]/ARB11500.1/0.0
P7_024 – Putative baseplate assembly protein	22,524	23267	+	247	GTG	TAA	Putative baseplate assembly protein [*Pectobacterium* phage phiTE]/YP_007392694.1/0.0
P7_025 – Putative tail lysozyme	23,276	23809	+	177	ATG	TGA	Putative tail lysozyme [*Pectobacterium* phage vB_PatM_CB7]/ARB11502.1/1.24493e-126
P7_026 – Putative baseplate assembly protein	23,899	25383	+	494	ATG	TAA	Putative baseplate assembly protein [*Pectobacterium* phage vB_PatM_CB7]/ARB11503.1/0.0
P7_027 – Hypothetical protein	25,393	26037	+	214	ATG	TAG	Hypothetical protein HL10_gp027 [*Cronobacter* phage CR8]/YP_009042264.1/4.87442e-138
P7_028 – Putative tail-fiber protein	26,048	27508	+	486	ATG	TAA	Putative tail-fiber protein [*Pectobacterium* phage ZF40]/YP_007006976.1/3.06883e-155

P7_029 – Hypothetical protein	27,510	28154	+	214	ATG	TGA	Hypothetical protein F396_gp68 [*Pectobacterium* phage ZF40]/YP_007006977.1/3.93737e-08
P7_030 – Hypothetical protein	28,181	28516	+	111	ATG	TGA	Hypothetical protein CL97_gp029 [*Cronobacter* phage CR9]/YP_009014991.1/2.24539e-58
P7_031 – Putative membrane protein	28,513	28980	+	155	ATG	TAG	Putative membrane protein [*Pectobacterium* phage vB_PatM_CB7]/ARB11508.1/8.09905e-46
P7_032 – Hypothetical protein	28,992	29255	+	87	ATG	TAA	Hypothetical protein CL97_gp031 [*Cronobacter* phage CR9]/YP_009014993.1/2.08249e-47
P7_033 – Putative tail fiber protein 2	29,268	29528	+	86	GTG	TAA	Putative tail fiber protein 2 [*Cronobacter* phage CR9]/YP_009014994.1/3.45954e-31
P7_034 – Hypothetical protein	29,563	29,892	-	109	GTG	TGA	Hypothetical protein P1A145kb_p036 [Pectobacterium phage DU_PP_I]/ATS93436.1/ 1.62917e-65
P7_035 – Hypothetical protein	30,009	31,226	-	405	ATG	TGA	Hypothetical protein P1A145kb_p037 [*Pectobacterium* phage DU_PP_I]/ ATS93437.1/ 0.0
P7_036 – Hypothetical protein	31,312	31,497	-	61	ATG	TAA	Hypothetical protein P1A145kb_p038 [*Pectobacterium* phage DU_PP_I]/ATS93438.1/2.04132e-23
P7_037 – Hypothetical protein	31,510	31,884	-	124	ATG	TAA	Hypothetical protein HL10_gp037 [*Cronobacter* phage CR8]/YP_009042274.1/2.92148e-88
P7_038 – Hypothetical protein	31,940	32,602	-	220	ATG	TGA	Hypothetical protein HL10_gp038 [*Cronobacter* phage CR8]/YP_009042275.1/3.71405e-162
P7_039 – Hypothetical protein	32,602	32,973	-	123	ATG	TGA	Hypothetical protein HL10_gp039 [*Cronobacter* phage CR8]/YP_009042276.1/5.02618e-85
P7_040 – Hypothetical protein	32,970	33,266	-	98	GTG	TGA	Hypothetical protein ADU18_0147 [*Cronobacter* phage PBES 02]/YP_009189008.1/1.06898e-69
P7_041 – Hypothetical protein	33,409	33,828	-	139	ATG	TGA	Hypothetical protein HL10_gp041 [*Cronobacter* phage CR8]/YP_009042278.1/3.66285e-102
P7_042 – Hypothetical protein	33,892	34,560	-	222	ATG	TAA	Hypothetical protein HL10_gp042 [*Cronobacter* phage CR8]/YP_009042279.1/2.17823e-116
P7_043 – Hypothetical protein	34,557	34,772	-	71	ATG	TGA	Hypothetical protein CR3_gp042 [*Cronobacter* phage CR3]/YP_006383057.1/7.20643e-46
P7_044 – Hypothetical protein	34,774	35,103	-	109	ATG	TGA	Hypothetical protein CR3_gp043 [*Cronobacter* phage CR3]/YP_006383058.1/1.83337e-78
P7_045 – Hypothetical protein	35,103	35,393	-	96	ATG	TAA	Hypothetical protein CR3_gp044 [*Cronobacter* phage

							CR3]/YP_006383059.1/7.56404e-64
P7_046 – Hypothetical protein	35,395	35,628	-	77	ATG	TAA	Hypothetical protein ADU18_0154 [*Cronobacter* phage PBES 02]/YP_009189015.1/3.89025e-51
P7_047 – Hypothetical protein	35,612	36,028	-	138	ATG	TGA	Hypothetical protein HL10_gp047 [*Cronobacter* phage CR8]/YP_009042284.1/2.71618e-93
P7_048 – Hypothetical protein	36,018	36,365	-	115	ATG	TAA	Hypothetical protein CR3_gp046 [*Cronobacter* phage CR3]/YP_006383061.1/2.63931e-75
P7_049 – Hypothetical protein	36,376	36,597	-	73	ATG	TAA	Hypothetical protein CR3_gp048 [*Cronobacter* phage CR3]/YP_006383063.1/8.40211e-51
P7_050 – Hypothetical protein	36,591	37,265	-	224	ATG	TGA	Hypothetical protein OMEGA_50 [*Klebsiella* phage vB_KaeM_KaOmega]/QEG12118.1/ 3.90877e-165
P7_051 – Hypothetical protein	37,274	37,522	-	82	ATG	TGA	Hypothetical protein CL97_gp051 [*Cronobacter* phage CR9]/YP_009015013.1/2.88901e-38
P7_052 – Hypothetical protein	37,592	37,759	-	55	ATG	TGA	Hypothetical protein CR3_gp051 [*Cronobacter* phage CR3]/YP_006383066.1/2.15183e-22
P7_053 – Hypothetical protein	37,761	38,045	-	94	TTG	TGA	Hypothetical protein CL97_gp053 [*Cronobacter* phage CR9]/YP_009015015.1/v
P7_054 – Hypothetical protein	38,082	38,483	-	133	ATG	TAA	Hypothetical protein CL97_gp055 [*Cronobacter* phage CR9]/YP_009015017.14.3731e-73
P7_055 – Coil containing protein	38,545	40,551	-	668	ATG	TAA	Coil containing protein [*Vibrio* phage 1.170.O._10N.261.52.C3]/AUR92199.1/0.0
P7_056 – Hypothetical protein	40,584	40,865	-	93	ATG	TAA	Hypothetical protein CR3_gp056 [*Cronobacter* phage CR3]/YP_006383071.1/5.96775e-63
P7_057 – Hypothetical protein	40,855	41,046	-	63	ATG	TGA	Hypothetical protein CR3_gp057 [*Cronobacter* phage CR3]/YP_006383072.1/1.07089e-40
P7_058 – Hypothetical protein	41,036	41,203	-	55	ATG	TAA	Hypothetical protein ADU18_0168 [*Cronobacter* phage PBES 02]/YP_009189029.1/1.29186e-33
P7_059 – Hypothetical protein	41,204	41,398	-	64	ATG	TGA	Hypothetical protein CR3_gp058 [*Cronobacter* phage CR3]/YP_006383073.1/4.32424e-35
P7_060 – Putative restriction endonuclease	41,493	42,002	-	169	ATG	TAA	Putative restriction endonuclease [*Pseudomonas* phage vB_PsyM_KIL1]/YP_009276041.1/ 6.25104e-33
P7_061 – DNA polymerase	42,031	44,658	-	875	ATG	TAG	DNA polymerase [*Vibrio* phage 11895-B1]/YP_007673567.1/1.12819e-104
P7_062 – Hypothetical protein	44,675	44,857	-	60	ATG	TAG	Hypothetical protein CR3_gp061 [*Cronobacter* phage

							CR3]/YP_006383076.1/6.99818e-21
P7_063	44,902	45,117	-	71	ATG	TAA	
P7_064 – Hypothetical protein	45,110	45,310	-	66	GTG	TAA	Hypothetical protein [*Siphoviridae* sp.]/ QHJ81725.1/ 2.47252e-16
P7_065 – Hypothetical protein	45,307	45,516	-	69	ATG	TGA	Hypothetical protein P1A145kb_p055 [*Pectobacterium* phage DU_PP_I]/ATS93455.1/ 4.40208e-35
P7_066 – Hypothetical protein	45,509	45,895	-	128	ATG	TAA	Hypothetical protein phiTE_014 [Pectobacterium phage phiTE]/YP_007392476.1/7.50715e-39
P7_067 – Hypothetical protein	45,896	46,096	-	66	ATG	TGA	Hypothetical protein P1A145kb_p058 [*Pectobacterium* phage DU_PP_I]/ATS93458.1/ 9.61796e-16
P7_068 – Hypothetical protein	46,096	46,287	-	63	ATG	TAA	
P7_069 – Hypothetical protein	46,287	46,655	-	122	ATG	TAA	Hypothetical protein P1A145kb_p059 [*Pectobacterium* phage DU_PP_I]/ATS93459.1/ 5.66509e-50
P7_070 – Hypothetical protein	46,652	46,930	-	92	ATG	TGA	Hypothetical protein CL97_gp070 [*Cronobacter* phage CR9]/YP_009015032.1/9.00456e-35
P7_071 – Hypothetical protein	46,927	48,135	-	402	ATG	TGA	Hypothetical protein phiTE_019 [*Pectobacterium* phage phiTE]/YP_007392481.1/0.0
P7_072 – Hypothetical protein	48,176	48,289	-	37	ATG	TAA	Hypothetical protein GAP31_094 [*Cronobacter* phage vB_CsaM_GAP31]/YP_006986929.1/ 2.13354e-09
P7_073 – Hypothetical protein	48,429	48,563	-	44	ATG	TAG	Hypothetical protein OMEGA_71 [*Klebsiella* phage vB_KaeM_KaOmega]/QEG12139.1/ 8.56666e-16
P7_074 – Hypothetical protein	48,556	48,738	-	60	ATG	TAA	Hypothetical protein CB7_65 [*Pectobacterium* phage vB_PatM_CB7]/ARB11539.1/2.40631e-33
P7_075 – Hypothetical protein	48,735	48,914	-	59	ATG	TGA	Hypothetical protein P12B145kb_p063 [Pectobacterium phage DU_PP_IV]/ATS93779.1/ 5.58981e-34
P7_076 – Hypothetical protein	48,911	49,252	-	113	ATG	TGA	Hypothetical protein CR3_gp071 [*Cronobacter* phage CR3]/YP_006383086.1/3.47036e-63
P7_077 – Hypothetical protein	49,249	49,521	-	90	ATG	TGA	Hypothetical protein CR3_gp072 [*Cronobacter* phage CR3]/YP_006383087.1/4.55827e-62
P7_078 – Hypothetical protein	49,518	49,703	-	61	ATG	TGA	Hypothetical protein CB7_68 [*Pectobacterium* phage

							vB_PatM_CB7] ARB11542.1/4.13594e-23
P7_079 – Hypothetical protein	49,700	49,909	-	69	ATG	TGA	Hypothetical protein P12B145kb_p067 [*Pectobacterium* phage DU_PP_IV]/ATS93783.1/ 1.7404e-38
P7_080 – Hypothetical protein	49,902	50,090	-	62	ATG	TGA	Hypothetical protein ADU18_0190 [*Cronobacter* phage PBES 02]/YP_009189050.1/3.64621e-38
P7_081	50,087	50,236	-	49	ATG	TGA	
P7_082 – Hypothetical protein	50,233	50,535	-	100	ATG	TGA	Hypothetical protein HL10_gp076 [*Cronobacter* phage CR8]/YP_009042313.1/9.7361e-67
P7_083 – Putative primase/helicase	50,626	52,719	-	697	GTG	TAA	Putative primase/helicase [*Pectobacterium* phage vB_PatM_CB7]/ARB11547.1/0.0
P7_084 – Hypothetical protein	52,716	52,883	-	55	ATG	TGA	Hypothetical protein phiTE_024 [*Pectobacterium* phage phiTE]/YP_007392486.1/4.09509e-33
P7_085 – Hypothetical protein	52,883	53,158	-	91	ATG	TGA	Hypothetical protein CR3_gp078 [*Cronobacter* phage CR3]/YP_006383093.1/5.79914e-61
P7_086 – Hypothetical protein	53,194	53,388	-	64	GTG	TAA	Hypothetical protein CR3_gp079 [*Cronobacter* phage CR3]/YP_006383094.1/6.50315e-34
P7_087 – Hypothetical protein	53,561	53,779	-	72	ATG	TGA	Hypothetical protein HL10_gp082 [*Cronobacter* phage CR8]/YP_009042319.1/4.47027e-35
P7_088 – Hypothetical protein	53,776	54,075	-	99	GTG	TGA	Hypothetical protein HL10_gp083 [*Cronobacter* phage CR8]/YP_009042320.1/8.04686e-65
P7_089 – Hypothetical protein	54,053	54,217	-	54	ATG	TAG	Hypothetical protein HL10_gp084 [*Cronobacter* phage CR8]/YP_009042321.1/3.81707e-29
P7_090 – Hypothetical protein	54,210	54,623	-	137	ATG	TGA	Hypothetical protein P1A145kb_p077 [*Pectobacterium* phage DU_PP_I]/ATS93477.1/ 1.11995e-54
P7_091 – Hypothetical protein	54,616	54,927	-	103	ATG	TAA	Hypothetical protein CR3_gp083 [*Cronobacter* phage CR3]/YP_006383098.1/4.48323e-19
P7_092 – Putative helicase	54,924	56,285	-	453	ATG	TGA	Putative helicase [*Pectobacterium* phage vB_PatM_CB7]/ARB11554.1/0.0
P7_093 – Hypothetical protein	56,285	56,470	-	61	ATG	TAA	Hypothetical protein CR3_gp085 [*Cronobacter* phage CR3]/YP_006383100.1/2.16909e-38
P7_094 – Hypothetical protein	56,480	56,668	-	62	ATG	TAA	Hypothetical protein CR3_gp086 [*Cronobacter* phage CR3]/YP_006383101.1/1.57498e-39

P7_095 – Putative cell wall hydrolase	56,665	57,198	-	177	ATG	TGA	Putative cell wall hydrolase [*Cronobacter* phage CR8]/YP_009042327.1/4.95509e-132
P7_096 – Hypothetical protein	57,248	57,643	-	131	TTG	TGA	Hypothetical protein HL10_gp091 [*Cronobacter* phage CR8]/YP_009042328.1/1.43873e-95
P7_097 – Putative phosphatase	57,621	58,163	-	180	GTG	TGA	Putative phosphatase [*Cronobacter* phage CR8]/YP_009042329.1/2.23655e-134
P7_098 – Hypothetical protein	58,153	58,365	-	70	ATG	TAA	Hypothetical protein HL10_gp093 [*Cronobacter* phage CR8]/YP_009042330.1/1.232e-47
P7_99 – Hypothetical protein	58,374	58,634	-	86	GTG	TAG	Hypothetical protein P12B145kb_p088 [*Pectobacterium* phage DU_PP_IV]/ATS93804.1/ 5.21569e-57
P7_100 – Hypothetical protein	58,624	59,001	-	125	ATG	TGA	Hypothetical protein CR3_gp092 [*Cronobacter* phage CR3]/YP_006383107.1/4.01818e-89
P7_101 – Hypothetical protein	58,985	59,446	-	153	ATG	TGA	Hypothetical protein CR3_gp093 [*Cronobacter* phage CR3]/YP_006383108.1/2.16119e-113
P7_102 – Hypothetical protein	59,490	59,678	-	62	ATG	TAA	Hypothetical protein CR3_gp094 [*Cronobacter* phage CR3]/YP_006383109.1/3.23746e-40
P7_103 – Hypothetical protein	59,678	60,001	-	107	ATG	TAA	Hypothetical protein CR3_gp095 [*Cronobacter* phage CR3]/YP_006383110.1/3.21327e-76
P7_104 – Hypothetical protein	59,988	60,206	-	72	ATG	TAA	Hypothetical protein HL10_gp099 [*Cronobacter* phage CR8]/YP_009042336.1/7.23943e-46
P7_105 – Hypothetical protein	60,211	60,330	-	39	ATG	TAA	Hypothetical protein [*Klebsiella* phage 31]/QGH73737.1/3.21895e-05
P7_106 – Hypothetical protein	60,327	60,671	-	114	ATG	TGA	Hypothetical protein P1A145kb_p093 [*Pectobacterium* phage DU_PP_I]/ATS93493.1/ 6.21485e-80
P7_107 – Hypothetical protein	60,668	61,399	-	243	ATG	TGA	Hypothetical protein HL10_gp101 [*Cronobacter* phage CR8]/YP_009042338.1/7.77034e-180
P7_108 – Hypothetical protein	61,396	61,599	-	67	ATG	TGA	Hypothetical protein HL10_gp102 [*Cronobacter* phage CR8]/YP_009042339.1/5.21738e-43
P7_109 – Hypothetical protein	61,586	61,831	-	81	ATG	TGA	Hypothetical protein phiTE_049 [*Pectobacterium* phage phiTE]/YP_007392511.1/6.73825e-35
P7_110 – Hypothetical protein	61,831	63,078	-	415	ATG	TAA	Hypothetical protein HL10_gp104 [*Cronobacter* phage CR8]/YP_009042341.1/0.0
P7_111 – Hypothetical protein	63,088	63,351	-	87	ATG	TGA	Hypothetical protein CR3_gp102 [*Cronobacter* phage

							CR3]/YP_006383117.1/4.53105e-55
P7_112 – Hypothetical protein	63,351	63,626	-	91	ATG	TAA	Hypothetical protein HL10_gp106 [*Cronobacter* phage CR8]. YP_009042343.1/3.14803e-63
P7_113 – Hypothetical protein	63,619	63,885	-	88	ATG	TGA	Hypothetical protein HL10_gp107 [*Cronobacter* phage CR8]/YP_009042344.1/2.34427e-60
P7_114 – Hypothetical protein	63,882	64,115	-	77	ATG	TGA	Hypothetical protein CR3_gp105 [*Cronobacter* phage CR3]/YP_006383120.1/8.32593e-53
P7_115 – Hypothetical protein	64,112	64,405	-	97	ATG	TGA	Hypothetical protein ADU18_0226 [*Cronobacter* phage PBES 02]/YP_009189084.1/1.80711e-67
P7_116 – Hypothetical protein	64,398	64,925	-	175	ATG	TAA	Hypothetical protein CR3_gp107 [*Cronobacter* phage CR3]/YP_006383122.1/7.75898e-128
P7_117 – Hypothetical protein	64,927	66,090	-	387	ATG	TAA	Hypothetical protein P1A145kb_p102 [*Pectobacterium* phage DU_PP_I]/ATS93502.1/ 5.46836e-80
P7_118 – Putative metal dependent phosphohydrolase	66,207	66,761	+	184	ATG	TGA	Putative metal dependent phosphohydrolase [*Cronobacter* phage CR8]/YP_009042348.1/ 4.48152e-137
P7_119 – Hypothetical protein	66,758	66,928	+	56	ATG	TGA	Hypothetical protein CB7_105 [*Pectobacterium* phage vB_PatM_CB7]/ARB11579.1/7.40029e-36
P7_120 – Hypothetical protein	66,918	67,121	+	67	ATG	TGA	Hypothetical protein CB7_106 [*Pectobacterium* phage vB_PatM_CB7]/ARB11580.1/2.34988e-42
P7_121 – Hypothetical protein	67,118	67,648	+	176	ATG	TGA	Hypothetical protein CB7_107 [*Pectobacterium* phage vB_PatM_CB7]/ARB11581.1/5.54077e-126
P7_122 – Hypothetical protein	67,651	68,265	+	204	ATG	TAA	Hypothetical protein HL10_gp115 [*Cronobacter* phage CR8]/YP_009042352.1/1.13332e-149
P7_123 – Putative ATPase	68,331	69,566	+	411	ATG	TGA	Putative ATPase [*Pectobacterium* phage DU_PP_I]/ATS93508.1/0.0
P7_124 – Homing endonuclease	69,563	70,060	+	165	ATG	TGA	Homing endonuclease [*Pectobacterium* phage Arno162]/AZV02184.1/9.67054e-33
P7_125 – Hypothetical protein	70,127	71,683	+	518	ATG	TAA	Hypothetical protein HL10_gp117 [*Cronobacter* phage CR8]/YP_009042354.1/0.0
P7_126 – Hypothetical protein	71,692	72,069	+	125	ATG	TAG	Hypothetical protein CR3_gp114 [*Cronobacter* phage CR3]/YP_006383129.1/1.57151e-91
P7_127 – Hypothetical protein	72,310	72,552	+	80	ATG	TGA	Hypothetical protein HL10_gp119 [*Cronobacter* phage CR8]/YP_009042356.1/1.69008e-55

P7_128 – Hypothetical protein	72,586	72,747	+	53	TTG	TAA	Hypothetical protein phiTE_065 [*Pectobacterium* phage phiTE]/YP_007392527.1/1.10322e-24
P7_129 – Hypothetical protein	72,740	72,862	+	40	ATG	TGA	Hypothetical protein CB7_114 [*Pectobacterium* phage vB_PatM_CB7]/ARB11588.1/4.32896e-21
P7_130 – Hypothetical protein	72,859	73,206	+	115	ATG	TAA	Hypothetical protein HL10_gp120 [*Cronobacter* phage CR8]/YP_009042357.1/7.69664e-77
P7_131 – Hypothetical protein	73,187	73,345	+	52	GTG	TGA	Hypothetical protein ADU18_0241 [*Cronobacter* phage PBES 02]/YP_009189096.1/5.68486e-28
P7_132 – Hypothetical protein	73,342	73,446	+	34	ATG	TAA	Hypothetical protein P1A145kb_p118 [*Pectobacterium* phage DU_PP_I]/ATS93518.1/ 3.0016e-11
P7_133 – Hypothetical protein	73,446	73,646	+	66	ATG	TAA	Hypothetical protein P1A145kb_p119 [*Pectobacterium* phage DU_PP_I]/ATS93519.1| 6.67511e-41
P7_134 – Hypothetical protein	73,649	74,188	+	179	ATG	TAA	Hypothetical protein CR3_gp119 [*Cronobacter* phage CR3]/YP_006383134.1/1.15939e-122
P7_135 – Hypothetical protein	74,181	74,390	+	69	ATG	TAA	Hypothetical protein CR3_gp120 [*Cronobacter* phage CR3]/YP_006383135.1/8.22827e-41
P7_136 – Hypothetical protein	74,468	74,698	+	76	ATG	TAA	Hypothetical protein HL10_gp125 [*Cronobacter* phage CR8]/YP_009042362.1/1.22697e-47
P7_137 – Hypothetical protein	74,698	74,889	+	63	ATG	TGA	Hypothetical protein phiTE_074 [*Pectobacterium* phage phiTE]/YP_007392536.1/1.86316e-39
P7_138 – Hypothetical protein	74,886	75,116	+	76	ATG	TAA	Hypothetical protein CR3_gp124 [*Cronobacter* phage CR3]/YP_006383139.1/8.1512e-48
P7_139 – Hypothetical protein	75,118	75,393	+	91	ATG	TGA	Hypothetical protein CL97_gp142 [*Cronobacter* phage CR9]/YP_009015104.1/8.40247e-37
P7_140 – Hypothetical protein	75,390	75,647	+	85	ATG	TGA	Hypothetical protein CB7_122 [*Pectobacterium* phage vB_PatM_CB7]/ARB11596.1/2.7315e-58
P7_141 – Hypothetical protein	75,706	76,566	+	286	ATG	TGA	Hypothetical protein P1A145kb_p128 [*Pectobacterium* phage DU_PP_I]/ATS93528.1/0.0
P7_142 – Hypothetical protein	76,616	77,155	+	179	ATG	TGA	Hypothetical protein GAP32_355 [*Cronobacter* phage vB_CsaM_GAP32]/YP_006987460.1/ 5.27976e-25
P7_143	77,231	77,329	+	32	TTG	TAA	
P7_144	77,407	77,643	+	78	TTG	TAG	

P7_145	77,758	77,925	+	55	TTG	TAG	
P7_146	78,079	78,153	+	24	GTG	TAG	
P7_147 – Hypothetical protein	78,254	78,586	+	110	ATG	TAA	Hypothetical protein HL10_gp132 [*Cronobacter* phage CR8]/YP_009042369.1/2.40567e-78
P7_148	78,676	78,903	+	75	ATG	TAA	
P7_149 – Hypothetical protein	78,991	79,212	+	73	ATG	TAA	Hypothetical protein CR3_gp130 [*Cronobacter* phage CR3]/YP_006383145.1/2.79717e-46
P7_150 – Hypothetical protein	79,259	79,603	+	114	ATG	TAA	Hypothetical protein HL10_gp135 [*Cronobacter* phage CR8]/YP_009042372.1/8.21885e-82
P7_151 – Hypothetical protein	79,706	79,945	+	79	ATG	TAA	Hypothetical protein HL10_gp137 [*Cronobacter* phage CR8]/YP_009042374.1/1.60315e-53
P7_152 – Putative membrane protein	80,024	80,254	+	76	ATG	TGA	Putative membrane protein [*Pectobacterium* phage vB_PatM_CB7]/ARB11603.1/9.5241e-51
P7_153 – Hypothetical protein	80,331	80,711	+	126	ATG	TAA	Hypothetical protein CR3_gp134 [*Cronobacter* phage CR3]/YP_006383149.1/3.29077e-89
P7_154 – Hypothetical protein	80,828	80,989	+	53	GTG	TAG	Hypothetical protein CR3_gp135 [*Cronobacter* phage CR3]/YP_006383150.1/3.04955e-31
P7_155 – Hypothetical protein	81,075	81,461	+	128	ATG	TAA	Hypothetical protein CR3_gp136 [*Cronobacter* phage CR3]/YP_006383151.1/2.68237e-90
P7_156 – Hypothetical protein	81,561	81,782	+	73	ATG	TGA	Hypothetical protein ADU18_0263 [*Cronobacter* phage PBES 02]/YP_009189118.1/6.29731e-47
P7_157 – Hypothetical protein	81,785	81,919	+	44	ATG	TAA	Hypothetical protein CB7_133 [*Pectobacterium* phage vB_PatM_CB7]/ARB11607.1/1.09963e-25
P7_158 – Hypothetical protein	81,996	82,235	+	79	ATG	TAA	Hypothetical protein HL10_gp143 [*Cronobacter* phage CR8]/YP_009042380.1/2.51881e-48
P7_159 – Hypothetical protein	82,339	82,773	+	144	ATG	TAA	Hypothetical protein HL10_gp144 [*Cronobacter* phage CR8]/YP_009042381.1/2.06242e-105
P7_160 – Hypothetical protein	82,837	83,169	+	110	ATG	TGA	Hypothetical protein P1A145kb_p141 [*Pectobacterium* phage DU_PP_I]/ATS93541.1/1.06601e-26
P7_161 – Hypothetical protein	83,249	83,500	+	83	ATG	TAA	Hypothetical protein P12B145kb_p143 [*Pectobacterium* phage DU_PP_IV]/ATS93859.1/1.48146e-56
P7_162 – Hypothetical protein	83,575	83,937	+	120	ATG	TAA	Hypothetical protein OMEGA_161 [*Klebsiella* phage

							vB_KaeM_KaOmega]/ QEG12227.1/ 2.5286e-78
P7_163 – Hypothetical protein	84,007	84,213	+	68	ATG	TGA	Hypothetical protein CR3_gp141 [*Cronobacter* phage CR3]/YP_006383156.1/8.27422e-44
P7_164 – Hypothetical protein	84,210	84,389	+	59	ATG	TAA	Hypothetical protein P1A145kb_p143 [*Pectobacterium* phage DU_PP_I]/ATS93543.1/ 4.50296e-38
P7_165 – Hypothetical protein	84,455	84,760	+	101	ATG	TAA	Hypothetical protein CR3_gp143 [*Cronobacter* phage CR3]/YP_006383158.1/5.91089e-70
P7_166 – Hypothetical protein	84,747	84,902	+	51	ATG	TAG	Hypothetical protein OMEGA_164 [*Klebsiella* phage vB_KaeM_KaOmega]/QEG12230.1/ 2.06932e-30
P7_167 – Hypothetical protein	84,979	85,137	+	52	ATG	TGA	Hypothetical protein OMEGA_165 [*Klebsiella* phage vB_KaeM_KaOmega]/ QEG12231.1/7.7969e-33
P7_168 – Hypothetical protein	85,165	85,404	+	79	ATG	TAA	Hypothetical protein OMEGA_166 [*Klebsiella* phage vB_KaeM_KaOmega]/QEG12232.1/4.16483e-46
P7_169 – Hypothetical protein	85,473	85,727	+	84	ATG	TAA	Hypothetical protein CR3_gp145 [*Cronobacter* phage CR3]/YP_006383160.1/7.05758e-58
P7_170 – Hypothetical protein	85,816	86,187	+	123	ATG	TAA	Hypothetical protein HL10_gp151 [*Cronobacter* phage CR8]/YP_009042388.1/2.27803e-81
P7_171 – Hypothetical protein	86,363	86,755	+	130	ATG	TGA	Hypothetical protein ADU18_0276 [*Cronobacter* phage PBES 02]/YP_009189130.1/1.2566e-78
P7_172 – Hypothetical protein	86,916	87,380	+	154	GTG	TGA	Hypothetical protein CL97_gp167 [*Cronobacter* phage CR9]/YP_009015129.1/4.82306e-102
P7_173 – Hypothetical protein	87,459	87,710	+	83	ATG	TAA	Hypothetical protein ADU18_0278 [*Cronobacter* phage PBES 02]/YP_009189132.1/4.54318e-54
P7_174 – Hypothetical protein	87,747	87,971	+	74	ATG	TGA	Hypothetical protein CR3_gp151 [*Cronobacter* phage CR3]/YP_006383166.1/7.89093e-49
P7_175 – Hypothetical protein	87,968	88,162	+	64	ATG	TGA	Hypothetical protein CL97_gp170 [*Cronobacter* phage CR9]/YP_009015132.1/2.56643e-37
P7_176 – Hypothetical protein	88,159	88,473	+	104	ATG	TAA	Hypothetical protein HL10_gp158 [*Cronobacter* phage CR8]/YP_009042395.1/1.18601e-56
P7_177 – Hypothetical protein	88,481	88,696	+	71	GTG	TGA	Hypothetical protein OMEGA_176 [*Klebsiella* phage vB_KaeM_KaOmega]/ QEG12364.1/ 2.61518e-38
P7_178 – Hypothetical protein	88,972	89,343	+	123	ATG	TAA	Hypothetical protein BI014_gp179 [*Klebsiella* phage PKO111]/YP_009289580.1/2.96579e-20
P7_179 – Hypothetical protein	89,420	89,746	+	108	ATG	TAA	Hypothetical protein CR3_gp156 [*Cronobacter* phage

							CR3]/YP_006383171.1/1.09275e-28
P7_180 – Hypothetical protein	89,812	90,057	+	81	ATG	TAA	Hypothetical protein CR3_gp157 [*Cronobacter* phage CR3]/YP_006383172.1/1.51417e-51
P7_181 – Hypothetical protein	90,934	91,050	+	38	GTG	TGA	Hypothetical protein OMEGA_182 [*Klebsiella* phage vB_KaeM_KaOmega]/QEG12366.1/8e-12
P7_182 – Hypothetical protein	91,245	91,532	-	95	GTG	TAG	Hypothetical protein OMEGA_183 [*Klebsiella* phage vB_KaeM_KaOmega]/QEG12367.1/2e-16
P7_183 – Hypothetical protein	91,641	91,862	-	73	GTG	TGA	Hypothetical protein CR3_gp159 [*Cronobacter* phage CR3]/YP_006383174.1/3.3979e-45
P7_184 – Hypothetical protein	91,931	92,143	-	70	ATG	TGA	Hypothetical protein P1A145kb_p154 [*Pectobacterium* phage DU_PP_I]/ATS93554.1/4.69745e-45
P7_185 – Hypothetical protein	92,140	92,505	-	121	GTG	TGA	Hypothetical protein P1A145kb_p155 [*Pectobacterium* phage DU_PP_I]/ATS93555.1/7.07631e-73
P7_186	92,515	92,730	-	71	ATG	TAA	
P7_187	92,805	93,008	-	67	ATG	TAA	
P7_188	93,005	93,496	-	163	ATG	TGA	
P7_189 – Hypothetical protein	93,500	93,751	-	83	ATG	TAG	Hypothetical protein phiTE_105 [*Pectobacterium* phage phiTE]/YP_007392567.1/7.2736e-34
P7_190 – Hypothetical protein	93,744	93,980	-	78	ATG	TAA	Hypothetical protein AXJ13_gp047 [*Achromobacter* phage JWF]/YP_009224053.1/7.94773e-06
P7_191 – Hypothetical protein	94,012	94,491	-	159	GTG	TGA	Hypothetical protein [*Pectobacterium odoriferum*]/WP_155278028.1/3e-19
P7_192	94,725	94,946	-	73	ATG	TGA	
P7_193	94,943	95,110	-	55	ATG	TGA	
P7_194 – Super infection exclusion gene product 17	95,094	95,423	-	109	ATG	TAA	Superinfection exclusion protein [*Enterobacteria* phage ST104]/YP_006372.1/7.14567e-38
P7_195 – Hypothetical protein	95,444	95,629	-	61	ATG	TAA	Hypothetical protein HWB37_gp056 [*Raoultella* phage Ro1]/YP_009835730.1/3.66236e-16
P7_196 – Hypothetical protein	95,849	96,184	-	111	ATG	TGA	Hypothetical protein CL97_gp190 [*Cronobacter* phage CR9]/YP_009015152.1/5.76949e-75
P7_197 – Hypothetical protein	96,165	96,401	-	78	ATG	TAA	Hypothetical protein CR3_gp172 [*Cronobacter* phage CR3]/YP_006383187.1/1.28599e-52

P7_198 – Hypothetical protein	96,385	96,903	-	172	ATG	TAA	Hypothetical protein ADU18_0014 [*Cronobacter* phage PBES 02]/YP_009188880.1/1.35155e-118
P7_199 – Hypothetical protein	96,884	97,114	-	76	GTG	TAA	Hypothetical protein phiTE_114 [*Pectobacterium* phage phiTE]/YP_007392576.1/7.1424e-49
P7_200 – Hypothetical protein	97,032	97,361	-	109	ATG	TAA	Hypothetical protein CR3_gp175 [*Cronobacter* phage CR3]/YP_006383190.1/5.06974e-76
P7_201 – Hypothetical protein	97,361	97,588	-	75	GTG	TGA	Hypothetical protein CB7_161 [*Pectobacterium* phage vB_PatM_CB7]/ ARB11719.1/6.77321e-50
P7_202 – Hypothetical protein	97,585	97,824	-	79	ATG	TGA	Hypothetical protein phiTE_117 [*Pectobacterium* phage phiTE]/YP_007392579.1/8.70849e-53
P7_203 – Hypothetical protein	97,900	98,070	-	56	ATG	TAA	Hypothetical protein phiTE_118 [*Pectobacterium* phage phiTE]/YP_007392580.1/4.11001e-35
P7_204 – Hypothetical protein	98,013	98,519	-	168	GTG	TGA	Hypothetical protein CR3_gp177 [*Cronobacter* phage CR3]/YP_006383192.1/1.41713e-125
P7_205 – Hypothetical protein	98,503	98,724	-	73	ATG	TAA	Hypothetical protein CR3_gp178 [*Cronobacter* phage CR3]/YP_006383193.1/1.46553e-43
P7_206 – Hypothetical protein	98,721	99,059	-	112	ATG	TGA	Hypothetical protein CL97_gp196 [*Cronobacter* phage CR9]/YP_009015158.1/6.94864e-38
P7_207 – Hypothetical protein	99,195	99,404	-	69	GTG	TAA	Hypothetical protein P1A145kb_p171 [*Pectobacterium* phage DU_PP_I]/ATS93571.1/2.01583e-44
P7_208 – Hypothetical protein	99,386	99,682	-	98	ATG	TAA	Hypothetical protein P1A145kb_p172 [*Pectobacterium* phage DU_PP_I]/ATS93572.1/2.52033e-69
P7_209 – Hypothetical protein	99,684	99,947	-	87	ATG	TGA	Hypothetical protein HL10_gp185 [*Cronobacter* phage CR8]/YP_009042422.1/2.10429e-58
P7_210 – Hypothetical protein	99,959	100,249	-	96	ATG	TAA	Hypothetical protein CR3_gp183 [*Cronobacter* phage CR3]/YP_006383198.1/8.08944e-64
P7_211 – Hypothetical protein	100,249	100,422	-	57	GTG	TGA	Hypothetical protein OMEGA_215 [*Klebsiella* phage vB_KaeM_KaOmega]/QEG12277.1/6.98426e-25
P7_212 – Hypothetical protein	100,477	100,929	-	150	ATG	TGA	Hypothetical protein CR3_gp185 [*Cronobacter* phage CR3]/YP_006383200.1/1.17717e-91
P7_213 – Hypothetical protein	100,922	101,335	-	137	ATG	TAA	Hypothetical protein OMEGA_217 [*Klebsiella* phage vB_KaeM_KaOmega]/QEG12279.1/2.68562e-78
P7_214 – Hypothetical protein	101,313	101,786	-	157	ATG	TAA	Hypothetical protein OMEGA_218 [*Klebsiella* phage

							vB_KaeM_KaOmega]/QEG12280.1/5.94157e-113
P7_215 – Hypothetical protein	101,789	102,304	-	171	ATG	TGA	Hypothetical protein OMEGA_219 [*Klebsiella* phage vB_KaeM_KaOmega]/QEG12281.1/4.53869e-124
P7_216 – Hypothetical protein	102,273	102,698	-	141	ATG	TGA	Hypothetical protein OMEGA_220 [*Klebsiella* phage vB_KaeM_KaOmega]/QEG12282.1/8.81039e-97
P7_217 – Hypothetical protein	102,745	103,188	-	147	ATG	TAA	Hypothetical protein HL10_gp193 [*Cronobacter* phage CR8]/YP_009042430.1/6.01925e-106
P7_218 – Hypothetical protein	103,394	103,987	-	197	ATG	TAA	Hypothetical protein P1A145kb_p182 [*Pectobacterium* phage DU_PP_I]/ATS93582.1/1.57567e-144
P7_219 – Hypothetical protein	103,989	104,249	-	86	GTG	TAA	Hypothetical protein P1A145kb_p183 [*Pectobacterium* phage DU_PP_I]/ATS93583.1/3.17383e-58
P7_220 – Hypothetical protein	104,259	104,735	-	158	ATG	TAG	Hypothetical protein ADU18_0033 [*Cronobacter* phage PBES 02]/YP_009188898.1/1.12506e-116
P7_221 – Putative nucleotidyltransferase	104,747	105,871	-	374	ATG	TGA	Putative nucleotidyltransferase [*Pectobacterium* phage DU_PP_I]/ATS93585.1/0.0
P7_222 – Hypothetical protein	105,959	106,360	-	133	ATG	TAA	Hypothetical protein HL10_gp199 [*Cronobacter* phage CR8]/YP_009042436.1/3.10113e-90
P7_223 – Hypothetical protein	106,360	106,761	-	133	ATG	TAA	Hypothetical protein HL10_gp200 [*Cronobacter* phage CR8]/YP_009042437.11.13379e-93
P7_224 – Hypothetical protein	106,758	107,057	-	99	ATG	TGA	Hypothetical protein OMEGA_228 [*Klebsiella* phage vB_KaeM_KaOmega]/QEG12290.1/6.47397e-61
P7_225 – Hypothetical protein	107,059	107,514	-	151	GTG	TAA	Hypothetical protein ADU18_0038 [*Cronobacter* phage PBES 02]/YP_009188903.1/2.00642e-109
P7_226 – Hypothetical protein	107,511	107,741	-	76	ATG	TGA	Hypothetical protein CL97_gp216 [*Cronobacter* phage CR9]/YP_009015178.1/3.99474e-42
P7_227 – tRNA nucleotidyl transferase	107,797	109,113	-	438	ATG	TAG	TRNA nucleotidyl transferase [*Pectobacterium* phage phiTE]/YP_007392598.1/0.0
P7_228 – Hypothetical protein	109,110	109,274	-	54	ATG	TGA	Hypothetical protein G377_gp017 [*Escherichia phage* phAPEC8]/YP_007348617.1/2.07964e-17
P7_229 – Hypothetical protein	109,271	109,603	-	110	ATG	TGA	Hypothetical protein OMEGA_233 [*Klebsiella* phage vB_KaeM_KaOmega]/QEG12295.1/2.52429e-70
P7_230 – Putative DNA methyltransferase	109,687	110,199	-	170	ATG	TAA	Putative DNA methyltransferase [*Cronobacter* phage CR9]/YP_009015182.1/5.70271e-119

P7_231 – Putative ATP-dependent protease subunit	110,174	111,151	-	325	ATG	TGA	Putative ATP-dependent protease subunit [*Pectobacterium* phage DU_PP_I]/ATS93595.1/6.28291e-152
P7_232 – Hypothetical protein	111,151	111,627	-	158	ATG	TAA	Hypothetical protein OMEGA_236 [*Klebsiella* phage vB_KaeM_KaOmega]/QEG12298.1/1.69238e-81
P7_233 – Hypothetical protein	111,638	111,952	-	104	GTG	TAA	Hypothetical protein phiTE_143 [*Pectobacterium* phage phiTE]/YP_007392605.1/5.42828e-73
P7_234 – Hypothetical protein	111,936	112,553	-	205	GTG	TAA	Hypothetical protein P1A145kb_p198 [*Pectobacterium* phage DU_PP_I]/ATS93598.1/1.109e-150
P7_235 – Hypothetical protein	112,540	113,031	-	163	GTG	TAG	Hypothetical protein ADU18_0048 [*Cronobacter* phage PBES 02]/YP_009188912.1/4.5066e-121
P7_236 – PhoH family protein	113,139	113,885	-	248	ATG	TAA	PhoH family protein [*Cronobacter* phage PBES 02]/YP_009188913.1/0.0
P7_237 – Peptidoglycan binding domain-containing protein	113,930	114,523	-	197	GTG	TAA	Peptidoglycan binding domain-containing protein [*Cronobacter* phage PBES 02]/YP_009188914.1/5.00332e-147
P7_238 – Hypothetical protein	114,533	114,946	-	137	ATG	TAA	Hypothetical protein HL10_gp214 [*Cronobacter* phage CR8]/YP_009042451.1/1.28382e-93
P7_239 – Glutaredoxin-related protein	114,957	115,244	-	95	ATG	TAA	Glutaredoxin-related protein [*Cronobacter* phage CR8]/YP_009042452.1/3.4474e-67
P7_240 – Hypothetical protein	115,244	115,423	-	59	ATG	TGA	Hypothetical protein CR3_gp213 [*Cronobacter* phage CR3]/YP_006383228.1/5.6143e-38
P7_241 – Putative ribonucleotide-diphosphate reductase subunit beta	115,492	116,622	-	376	ATG	TAA	Putative ribonucleotide-diphosphate reductase subunit bet [*Pectobacterium* phage DU_PP_I]/ATS93606.1/0.0
P7_242 – Hypothetical protein	116,687	116,857	-	56	ATG	TAG	Hypothetical protein HL10_gp219 [*Cronobacter* phage CR8]/YP_009042456.1/1.66934e-35
P7_243 – Hypothetical protein	116,854	117,507	-	217	ATG	TGA	Hypothetical protein P1A145kb_p208 [*Pectobacterium* phage DU_PP_I]/ATS93608.1/2.29826e-161
P7_244 – Ribonucleotide reductase subunit A	117,543	119,798	-	751	TTG	TAA	Ribonucleotide reductase subunit A [*Synechococcus* phage ACG-2014g]/YP_009133735.1/0.0
P7_245 – Hypothetical protein	119,817	120,047	-	76	ATG	TAA	Hypothetical protein CR3_gp217 [*Cronobacter* phage CR3]/YP_006383232.1/2.65507e-52

P7_246 – Hypothetical protein	120,050	120,544	-	164	ATG	TAA	Hypothetical protein CR3_gp218 [*Cronobacter* phage CR3]/YP_006383233.1/5.51909e-117
P7_247 – Putative thymidylate synthase	120,626	121,639	-	337	ATG	TAA	Putative thymidylate synthase [*Cronobacter* phage PBES 02]/YP_009188923.1/0.0
P7_248 – Hypothetical protein	121,654	121,836	-	60	ATG	TAA	Hypothetical protein CR3_gp220 [*Cronobacter* phage CR3]/YP_006383235.1/7.48878e-38
P7_249 – Hypothetical protein	121,836	122,447	-	203	ATG	TAA	Hypothetical protein HL10_gp225 [*Cronobacter* phage CR8]/YP_009042462.1/8.41073e-152
P7_250 – Hypothetical protein	122,434	123,093	-	219	ATG	TAA	Hypothetical protein CR3_gp222 [*Cronobacter* phage CR3]/YP_006383237.1/5.76036e-163
P7_251 – Hypothetical protein	123,093	123,401	-	102	ATG	TAA	Hypothetical protein HL10_gp227 [*Cronobacter* phage CR8]/YP_009042464.1/8.29941e-73
P7_252 – Putative DNA polymerase II	123,452	124,534	-	360	TTG	TAA	Putative DNA polymerase 2 [*Cronobacter* phage CR8]/YP_009042465.1/0.0
P7_253 – Homing endonuclease	124,668	125,030	-	120	ATG	TGA	Homing endonuclease [*Pectobacterium* phage Arno162]/AZV02184.1/1.56816e-10
P7_254 – Hypothetical protein	125,030	125,260	-	76	ATG	TGA	Hypothetical protein P1A145kb_p220 [*Pectobacterium* phage DU_PP_I]/ATS93620.1/7.87665e-52
P7_255 – Putative packaging and recombination endonuclease VII	125,253	125,765	-	170	GTG	TAA	Putative packaging and recombination endonuclease VII [*Cronobacter* phage CR3]/YP_006383241.1/4.84159e-124
P7_256 – Hypothetical protein	125,767	126,309	-	180	TTG	TAA	Hypothetical protein HL10_gp231 [*Cronobacter* phage CR8]/YP_009042468.1/1.79086e-131
P7_257 – Hypothetical protein	126,359	126,937	-	192	GTG	TAA	Hypothetical protein HL10_gp232 [*Cronobacter* phage CR8]/YP_009042469.1/3.84589e-142
P7_258 – Hypothetical protein	126,963	127,298	-	111	ATG	TGA	Hypothetical protein CR3_gp229 [*Cronobacter* phage CR3]/YP_006383244.1/8.37248e-79
P7_259 – Putative exodeoxyribonuclease	127,298	128,413	-	371	GTG	TAA	Putative exodeoxyribonuclease [*Pectobacterium* phage DU_PP_I]/ATS93625.1/0.0
P7_260 – HNH endonuclease	128,413	128,910	-	165	ATG	TAG	HNH endonuclease [*Pectobacterium* phage phiTE]/YP_007392634.1/1.43659e-114
P7_261 – HNH endonuclease	128,910	129,410	-	166	ATG	TGA	HNH endonuclease [*Pectobacterium* phage phiTE]/YP_007392635.1/4.00002e-118
P7_262 – Exonuclease	129,403	129,573	-	56	TTG	TAA	Exonuclease [Klebsiella phage

							vB_KpnM_BIS47]/YP_009832739.1/9.63665e-05
P7_263 – Hypothetical protein	129,563	130,114	-	183	ATG	TAA	Hypothetical protein P1A145kb_p229 [*Pectobacterium* phage DU_PP_I]/ ATS93629.1/2.12245e-126
P7_264 – Hypothetical protein	130,111	130,308	-	65	ATG	TGA	Hypothetical protein HL10_gp237 [*Cronobacter* phage CR8]/YP_009042474.1/1.37094e-43
P7_265 – Hypothetical protein	130,305	130,520	-	71	ATG	TGA	Hypothetical protein CR3_gp234 [*Cronobacter* phage CR3]/YP_006383249.1/3.01083e-42
P7_266 – Hypothetical protein	130,517	130,855	-	112	ATG	TGA	Hypothetical protein CR3_gp235 [*Cronobacter* phage CR3]/YP_006383250.1/5.68561e-79
P7_267 – Hypothetical protein	130,855	131,367	-	170	ATG	TGA	Hypothetical protein CL97_gp253 [*Cronobacter* phage CR9]/YP_009015215.1/6.16338e-78
P7_268 – Hypothetical protein	131,367	131,579	-	70	ATG	TGA	Hypothetical protein CL97_gp254 [*Cronobacter* phage CR9]/YP_009015216.1/5e-46
P7_269 – Hypothetical protein	131,587	131,793	-	68	ATG	TGA	Hypothetical protein HL10_gp243 [*Cronobacter* phage CR8]/YP_009042480.1/2.05776e-20
P7_270 – Hypothetical protein	131,790	131,996	-	68	GTG	TGA	Hypothetical protein CR3_gp240 [*Cronobacter* phage CR3]/YP_006383255.1/2.28445e-42
P7_271 – Hypothetical protein	131,993	132,496	-	167	ATG	TGA	Hypothetical protein CL97_gp257 [*Cronobacter* phage CR9]/YP_009015219.1/1.17699e-120
P7_272 – Hypothetical protein	132,595	132,876	-	93	ATG	TAA	Hypothetical protein CR3_gp242 [*Cronobacter* phage CR3]/YP_006383257.1/6.44547e-60
P7_273 – Hypothetical protein	132,948	133,265	-	105	ATG	TAA	Hypothetical protein CR3_gp243 [*Cronobacter* phage CR3]/YP_006383258.1/3.39889e-63
P7_274 – Putative pyrophosphatase	133,516	133,917	-	133	ATG	TAA	Putative pyrophosphatase [*Klebsiella* phage vB_KaeM_KaOmega]/QEG12336.1/2.72389e-93
P7_275 – Hypothetical protein	133,933	134,388	-	151	ATG	TGA	Hypothetical protein CL97_gp263 [*Cronobacter* phage CR9]/YP_009015225.1/1.37471e-44
P7_276 – DNA ligase	134,385	135,752	-	455	ATG	TGA	DNA ligase [*Pectobacterium* phage phiTE]/YP_007392649.1/0.0
P7_277 – Hypothetical protein	135,839	136,225	-	128	GTG	TGA	Hypothetical protein P1A145kb_p245 [*Pectobacterium* phage DU_PP_I]/ATS93645.1/5.87255e-89
P7_278 – Hypothetical protein	136,218	136,436	-	72	ATG	TAA	Hypothetical protein CR3_gp249 [*Cronobacter* phage CR3]/YP_006383264.1/2.48628e-47

P7_279 – Hypothetical protein	136,429	136,587	-	52	GTG	TGA	Hypothetical protein CR3_gp250 [*Cronobacter* phage CR3]/YP_006383265.1/9.41846e-29
P7_280 – Hypothetical protein	136,648	136,947	-	99	ATG	TGA	Hypothetical protein CR3_gp251 [*Cronobacter* phage CR3]/YP_006383266.1/3.6358e-70
P7_281 – Hypothetical protein	136,947	137,147	-	66	ATG	TAA	Hypothetical protein HL10_gp254 [*Cronobacter* phage CR8]/YP_009042491.1/2.74981e-43
P7_282 – Hypothetical protein	137,137	137,355	-	72	ATG	TAA	Hypothetical protein CR3_gp252 [*Cronobacter* phage CR3]/YP_006383267.1/6.71418e-37
P7_283 – Hypothetical protein	137,355	137,636	-	93	ATG	TGA	Hypothetical protein CR3_gp253 [*Cronobacter* phage CR3]/YP_006383268.1/2.0215e-62
P7_284 – Hypothetical protein	138,001	138,372	+	123	GTG	TGA	Hypothetical protein P1A145kb_p251 [*Pectobacterium* phage DU_PP_I]/ATS93651.1/4.10899e-87
P7_285 – Putative ribose-phosphate pyrophosphokinase	138,427	139,317	+	296	ATG	TGA	Putative ribose-phosphate pyrophosphokinase [*Pectobacterium* phage DU_PP_I]/ATS93652.1/0.0
P7_286 – HNH endonuclease	139,340	139,855	+	171	ATG	TGA	HNH endonuclease [*Pectobacterium* phage DU_PP_I]/ATS93653.1/6.39294e-126
P7_287 – Putative nicotinamide phosphoribosyl transferase	139,866	141,656	+	596	ATG	TAA	Putative nicotinamide phosphoribosyl transferase [*Klebsiella* phage vB_KpnM_KB57]/ YP_009187706.1/0.0
P7_288 – Hypothetical protein	141,702	142,031	+	109	ATG	TGA	Hypothetical protein CL97_gp274 [*Cronobacter* phage CR9]/YP_009015236.1/3.41951e-63
P7_289 – Hypothetical protein	142,091	142,342	+	83	ATG	TGA	Hypothetical protein CL97_gp275 [*Cronobacter* phage CR9]/YP_009015237.1/8.53623e-42
P7_290 – Hypothetical protein	142,339	142,575	+	78	TTG	TGA	Hypothetical protein CR3_gp260 [*Cronobacter* phage CR3]/YP_006383275.1/2.13976e-51
P7_291 – Hypothetical protein	142,523	142,744	+	73	GTG	TAA	Hypothetical protein OMEGA_291 [*Klebsiella* phage vB_KaeM_KaOmega]/ QEG12353.1/3e-10
P7_292 – Hypothetical protein	142,725	142,931	+	68	ATG	TGA	Hypothetical protein HL10_gp264 [*Cronobacter* phage CR8]/ YP_009042501.1/7e-22
tRNA-tRNA-Leu	143,151	143,229	+	None		CCA	
P7_293 – Hypothetical protein	143,301	143,675	+	124	ATG	TAA	Hypothetical protein CR3_gp261 [*Cronobacter* phage CR3]/YP_006383276.1/4.0428e-84
tRNA-tRNA-Tyr	143,842	143,929	+	None		CCA	

tRNA-tRNA-Ser	143,937	144,031	+	None		CCA	
P7_294 – Putative HNH endonuclease	144,061	144,495	+	144	GTG	TAA	Putative HNH endonuclease [*Erwinia* phage pEp_SNUABM_01]/YP_009851626.1/2.93001e-55
tRNA-tRNA-Asn	144,650	144,725	+	None		CCA	
P7_295 – Hypothetical protein	144,893	145,081	+	62	ATG	TAA	Hypothetical protein [*Siphoviridae* sp.]/DAM03608.1/2.98357e-30
tRNA-tRNA-Glu	145,395	145,472	+	None		CCA	
tRNA-tRNA-Asp	145,567	145,642	+	None		CCA	
P7_296 – Hypothetical protein	145,645	146,322	+	225	TTG	TAA	Hypothetical protein HL10_gp268 [*Cronobacter* phage CR8]/YP_009042503.1/4.81296e-145
tRNA-tRNA-Phe	146,326	146,399	+	None		CCA	
P7_297 – Hypothetical protein	146,509	146,880	+	123	ATG	TGA	Hypothetical protein CL97_gp280 [*Cronobacter* phage CR9]/YP_009015242.1/2.32122e-83
P7_298- – Hypothetical protein	146,880	147,194	+	104	ATG	TGA	Hypothetical protein CR3_gp265 [*Cronobacter* phage CR3]/YP_006383280.1/1.04177e-71

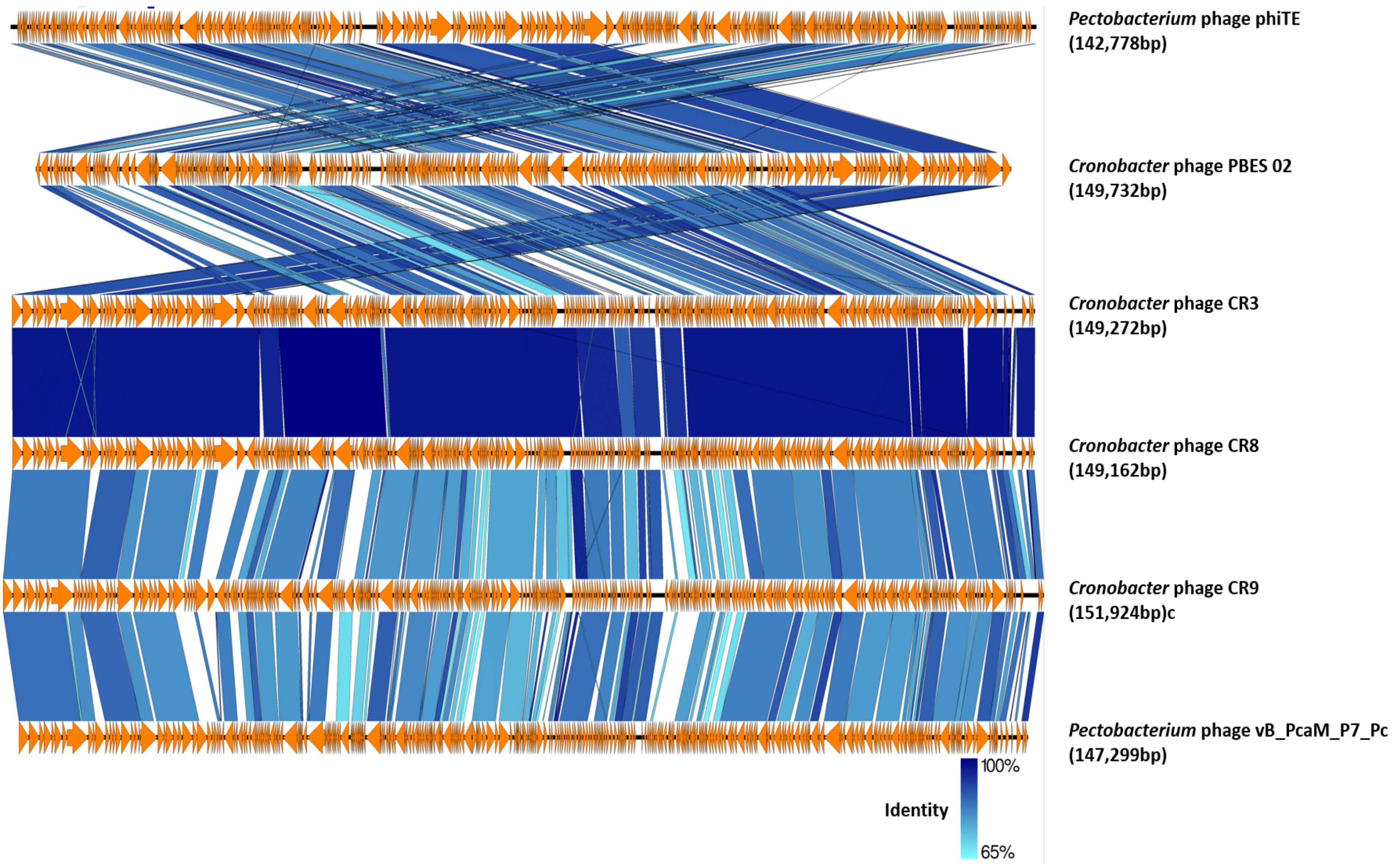

Figure S.2. **Genome comparison of *Pectobacterium* phage P7_Pc with five other members of the genus *Certrevirus***

The current annotations available at the Genbank were used for the comparisons employing BLASTN using EasyFig 2.2.5. Orange arrows show the gene locations and orientations in different phage genomes and blue lines between the genome maps illustrate the identity level.
